# Slow-wave sleep mediates the compensatory effects of acute moderate-intensity exercise on declarative memory deficits in sleep restriction

**DOI:** 10.1093/sleep/zsag235

**Published:** 2026-08-26

**Authors:** Emmanuel Frimpong, Melodee Mograss, Jocelyn Dautet-Plourde, Nicole Milbrath, Francois Pointeau, Manlu Xu, Ngoc Long Khang Nguyen, Tehila Zvionow, Mylene Aubertin-Leheudre, Louis Bherer, Véronique Pepin, Edwin Robertson, Arsenio Páez, Thien Thanh Dang-Vu

**Affiliations:** Sleep, Cognition & Neuroimaging Laboratory, Concordia University, Montreal, QC, Canada; Department of Health, Kinesiology & Applied Physiology, Concordia University, Montreal, QC, Canada; School of Health, Concordia University, Montreal, QC, Canada; Centre de Recherche de l’Institut Universitaire de Gériatrie de Montréal, Santé Québec Centre-Sud-de-l’île-de-Montréal Universitaire, Montréal, QC, Canada; Sleep, Cognition & Neuroimaging Laboratory, Concordia University, Montreal, QC, Canada; Department of Health, Kinesiology & Applied Physiology, Concordia University, Montreal, QC, Canada; School of Health, Concordia University, Montreal, QC, Canada; Department of Psychology, Concordia University, Montreal, QC, Canada; Sleep, Cognition & Neuroimaging Laboratory, Concordia University, Montreal, QC, Canada; Department of Health, Kinesiology & Applied Physiology, Concordia University, Montreal, QC, Canada; School of Health, Concordia University, Montreal, QC, Canada; Sleep, Cognition & Neuroimaging Laboratory, Concordia University, Montreal, QC, Canada; School of Health, Concordia University, Montreal, QC, Canada; Department of Psychology, Concordia University, Montreal, QC, Canada; Sleep, Cognition & Neuroimaging Laboratory, Concordia University, Montreal, QC, Canada; Department of Health, Kinesiology & Applied Physiology, Concordia University, Montreal, QC, Canada; School of Health, Concordia University, Montreal, QC, Canada; Centre de Recherche de l’Institut Universitaire de Gériatrie de Montréal, Santé Québec Centre-Sud-de-l’île-de-Montréal Universitaire, Montréal, QC, Canada; Sleep, Cognition & Neuroimaging Laboratory, Concordia University, Montreal, QC, Canada; School of Health, Concordia University, Montreal, QC, Canada; Department of Psychology, Concordia University, Montreal, QC, Canada; Sleep, Cognition & Neuroimaging Laboratory, Concordia University, Montreal, QC, Canada; School of Health, Concordia University, Montreal, QC, Canada; Department of Psychology, Concordia University, Montreal, QC, Canada; Sleep, Cognition & Neuroimaging Laboratory, Concordia University, Montreal, QC, Canada; Department of Health, Kinesiology & Applied Physiology, Concordia University, Montreal, QC, Canada; School of Health, Concordia University, Montreal, QC, Canada; Centre de Recherche de l’Institut Universitaire de Gériatrie de Montréal, Santé Québec Centre-Sud-de-l’île-de-Montréal Universitaire, Montréal, QC, Canada; Département des Sciences de l’activité physique, GRAPA, Université du Québec à Montréal, Montréal, QC, Canada; Centre de Recherche de l’Institut Universitaire de Gériatrie de Montréal, Santé Québec Centre-Sud-de-l’île-de-Montréal Universitaire, Montréal, QC, Canada; Department of Medicine and Centre de recherche de l’Institut de cardiologie de Montréal, Université de Montréal, Montréal, QC, Canada; Department of Health, Kinesiology & Applied Physiology, Concordia University, Montreal, QC, Canada; School of Health, Concordia University, Montreal, QC, Canada; Centre de recherche, CIUSSS du Nord-de l’Île-de-Montréal, Montréal, QC, Canada; School of Psychology and Neuroscience, University of Glasgow, Glasgow, United Kingdom; Sleep, Cognition & Neuroimaging Laboratory, Concordia University, Montreal, QC, Canada; Department of Health, Kinesiology & Applied Physiology, Concordia University, Montreal, QC, Canada; School of Health, Concordia University, Montreal, QC, Canada; Centre de Recherche de l’Institut Universitaire de Gériatrie de Montréal, Santé Québec Centre-Sud-de-l’île-de-Montréal Universitaire, Montréal, QC, Canada; Sleep, Cognition & Neuroimaging Laboratory, Concordia University, Montreal, QC, Canada; Department of Health, Kinesiology & Applied Physiology, Concordia University, Montreal, QC, Canada; School of Health, Concordia University, Montreal, QC, Canada; Centre de Recherche de l’Institut Universitaire de Gériatrie de Montréal, Santé Québec Centre-Sud-de-l’île-de-Montréal Universitaire, Montréal, QC, Canada; Department of Psychology, Concordia University, Montreal, QC, Canada

**Keywords:** sleep restriction, memory, discriminability index, acute exercise, slow-wave sleep, learning and memory

## Abstract

Insufficient sleep impairs cognition, particularly hippocampal-dependent memory. However, effective strategies for addressing these effects in humans remain limited. Physical exercise enhances slow-wave sleep (SWS) and improves memory performance, suggesting it may provide a physiologically grounded buffer against sleep-loss–related memory deficits. Here, we tested whether a single session of moderate-intensity exercise may protect long-term declarative memory following sleep restriction, and whether SWS mediates this effect. Eighty-eight healthy young adults (52% female; 24.0 ± 3.8 years) were randomly assigned to one of four groups based on exercise and sleep opportunity: average sleep (S_8_: 8.5 hours/night), sleep restriction (S5: 4.5 hours/night), exercise before average sleep (ExS_8_), and exercise before sleep restriction (ExS_5_). Participants completed either a 30-min evening moderate-intensity cycling (7: 00 p.m.; 55%–60% VO₂_max_) or seated rest prior to learning 80 face–name pairs (10:00 p.m.), followed by immediate and delayed recall (120 face-name pairs: 80 studied, 40 novel) after polysomnography-recorded sleep. We found a significant exercise × sleep interaction for delayed recall performance (discriminability index, *d′*; *p* = .033*, partial η^2^* = 0.056). The delayed recall *d′* of the ExS_5_ was significantly higher than the S_5_ (*p* = .028), but was comparable to S_8_ and ExS_8_ groups (*ps* > 0.05). The proportion of SWS mediated the association between exercise and delayed recall *d′* (*B* = 0.105, *SE* = 0.091, 95% bootstrap CI (0.073, 0.924)). These findings indicate that acute moderate-intensity exercise is associated with offsetting the negative impact of sleep restriction on long-term declarative memory in sleep restriction, an effect statistically mediated by SWS percentage.

## Teaser

Here, we demonstrate that a single session of moderate-intensity evening exercise performed before a night of sleep restriction was associated with better sleep-dependent memory consolidation and long-term declarative memory performance in healthy young adults. The proportion of Slow-wave sleep statistically mediated this effect, suggesting a mechanistic pathway through which acute exercise may support memory under sleep loss. These findings provide new insights and extend our prior work showing that acute high-intensity exercise partially attenuates sleep-restriction–related memory deficits. Collectively, the findings support acute exercise as a promising, practical countermeasure to sleep-loss-related cognitive impairment. Future studies should investigate whether these findings extend to at-risk populations due to occupational demands, circadian disruption, or sleep disorders.

## Graphical Abstract

**Graphical Abstract:**
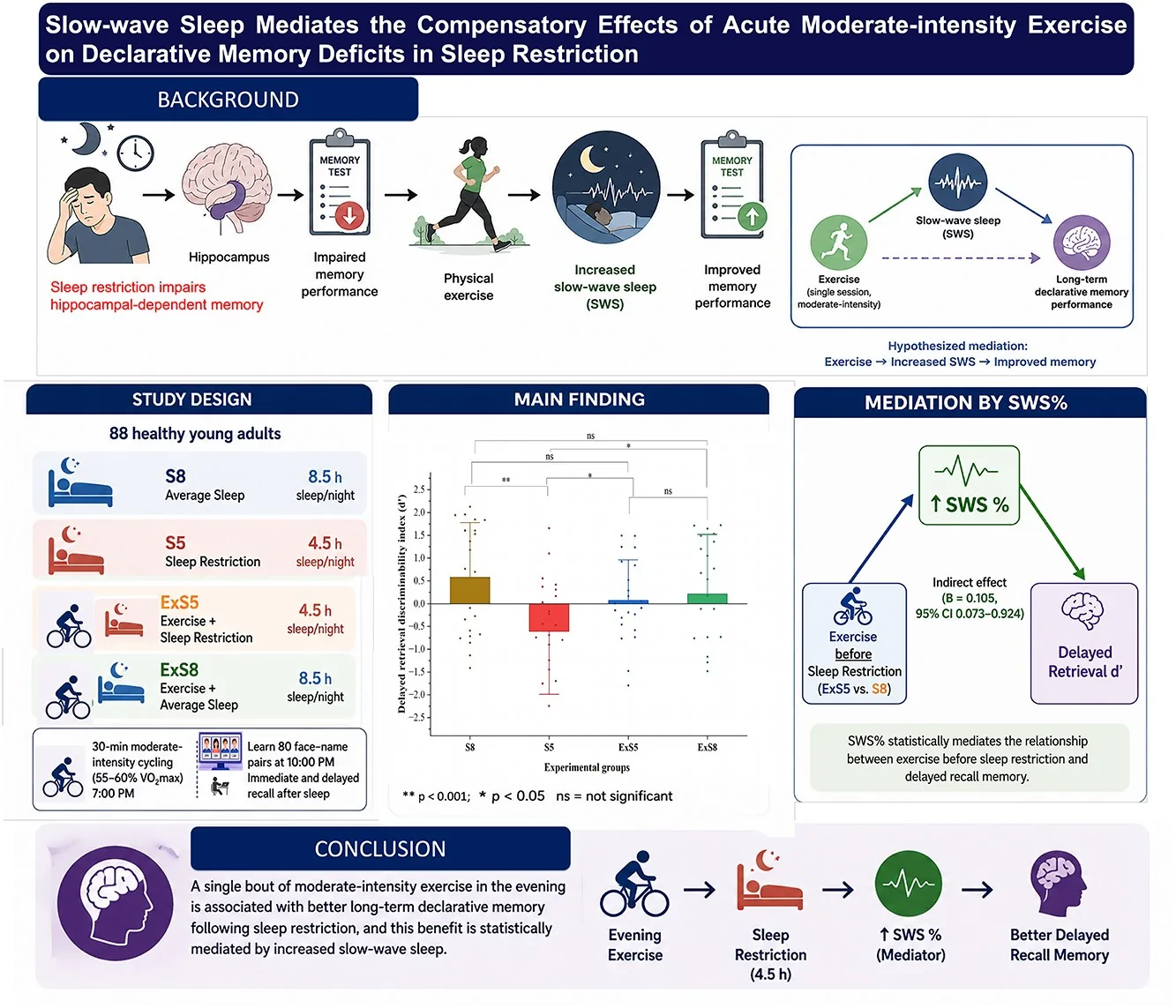

## Introduction

Adequate sleep is essential for memory processes, supporting the encoding, consolidation, and retrieval of newly learned information [1–4]. Compelling evidence indicate that sleep actively promotes the long-term consolidation of declarative memories [1–4]. In particular, non-rapid eye movement (NREM) sleep stage N3 (i.e. slow-wave sleep, SWS), promotes the reactivation of newly encoded hippocampal memories and their redistribution to neocortical networks for long-term storage [1–4]. Through this systems consolidation process, SWS stabilizes memory traces, rendering them less susceptible to interference and reducing forgetting at delayed retrieval, supporting memory retrieval that is increasingly independent of hippocampal engagement [5, 6]. Interventions that enhance SWS therefore provide a promising mechanism for strengthening memory consolidation and retention [7–10].

Conversely, insufficient sleep contributes to impairments in hippocampal-dependent memory, executive function, emotional regulation, and affective vulnerability [11, 12]. Sleep restriction—defined as sleep duration below the age-specific recommended range [13], impairs both brain and cognitive functioning [2, 14, 15]. Partial sleep restriction, particularly when it reduces early nocturnal sleep that is dominated by SWS, results in greater deficits in hippocampal-dependent declarative memory than restriction of the second-half night sleep, rich in REM sleep [2, 16]. These deficits not only reduce daytime memory performance but increase risk for anxiety and affective disorders, in which sleep disruption and impaired memory processes are core features [11, 12]. A recent meta-analysis found that even a single night of restricted sleep (3–6.5 hours) significantly impairs memory formation [17], resulting in memory deficits comparable to those induced by total sleep deprivation [17].

Insufficient sleep is pervasive globally [18], and the prevalence of sleep disorders such as insomnia is rising [19]. Yet, few interventions have been shown to effectively counteract the cognitive deficits caused by sleep loss or prevent memory impairment under acute sleep restriction [20]. In this context, physical exercise may constitute a compelling candidate.

Physical exercise induces increases in body temperature, autonomic and metabolic functions, important for promoting SWS through thermoregulatory and adenosine-mediated pathways [1, 21], as well as enhancing brain electrophysiology [22–25] to enhance memory consolidation [26–29]. Notably, sleep-dependent memory consolidation has been linked to brain oscillations during NREM sleep, namely the neocortical slow oscillations (SOs: <1 Hz), thalamocortical sleep spindles (11–16 Hz) and hippocampal sharp-wave ripples (~80–120 Hz) [3, 5, 30–32]. Slow oscillations occurring during SWS [10, 32, 33] and sleep spindles predominantly generated during stage N2 sleep, and also during SWS are key oscillatory mechanism required for offline memory consolidation [34–37]. Interventions that increase SWS and neuronal oscillations (e.g. SOs and spindle activity) during NREM sleep result in improved sleep-dependent memory consolidation [7–10]. Studies show that acute exercise results in robust increases in SWS [22–25] and brain oscillations, essential for sleep-dependent memory consolidation [25, 34, 38, 39]. Given that SWS is critical for hippocampal-dependent memory consolidation [1, 6], exercise may indirectly enhance memory by increasing SWS quantity or intensity.

In addition, acute moderate exercise also increases the levels of specific neurotrophic growth factors such as brain-derived neurotrophic factor (BDNF) and neuronal growth factor [40–43] in the hippocampus and other brain regions [43], which are closely linked to SWS generation and hippocampal plasticity [42]. The exercise-induced increases in neurotrophins modulate neurogenesis, long-term potentiation and synaptic plasticity, thereby stimulating learning and memory [40, 42–44]. Furthermore, the acute exercise-memory relationship may be moderated by factors such as the intensity, duration, and timing of exercise in relation to the memory processes [26–28]. When performed before memory encoding or during early memory consolidation [26, 28], acute exercise enhances long-term memory [45] by priming these molecular mechanisms involved in encoding and consolidation of newly acquired information [20, 46]. Meta-analytic reviews have shown that acute exercise performed before memory encoding [26, 28] or during early memory consolidation enhances memory [26].

Exercise is an effective non-pharmacological intervention for improving sleep quality [47], but its potential to mitigate the negative effects of sleep restriction on memory remains underinvestigated and the mechanisms by which exercise may buffer memory against sleep loss remain insufficiently understood [20, 48, 49]. Intriguingly, evidence suggests that exercise and sleep exert synergistic effects on memory processes [20, 50], supporting exercise as a promising intervention against memory impairments associated with insufficient or poor sleep. Cross-sectional findings indicate that higher physical activity levels may mitigate the cognitive deficits of poor sleep quality on memory in young and older adults [49, 51, 52]. Complementary evidence from animal research further shows that physical exercise before sleep deprivation can reverse the cognitive deficits induced by sleep loss [53].

However, important evidence gaps remain. Only one human experimental study has examined the compensatory effects of acute exercise (i.e. a single bout of exercise) on declarative memory in sleep restriction [48]. Our previous work demonstrated that a brief, remotely delivered and monitored, 15-minute high-intensity interval training (HIIT; at 85%–95% of age-predicted maximal heart rate) exercise intervention performed in the early evening before a night of restricted sleep (5.5 hours/night) was associated with partial attenuation of the detrimental effects of sleep loss on long-term declarative memory relative to an average sleep condition (8.5 hours/night) in healthy young adults [48]. However, that study lacked objective sleep assessments, such as polysomnography (PSG) or actigraphy, and objective monitoring of exercise intensity. Furthermore, it remains unclear whether this compensatory effect depends on exercise intensity, as prior evidence suggests that moderate-intensity cardiovascular exercise enhances the memory-promoting benefits of sleep [40, 41, 50, 54].

To address these gaps, we conducted a tightly controlled randomized laboratory experiment using individualized moderate-intensity exercise, objective PSG, and mediation modelling. Our aims were to: (1) investigate whether a single bout of evening moderate-intensity cardiovascular exercise performed prior to learning (encoding) could compensate for the detrimental effect of sleep restriction on long-term declarative memory compared to sleep restriction alone or an average night’s sleep opportunity in healthy young adults, and (2) test whether SWS percentage statistically mediates the effects of acute moderate-intensity exercise on long-term memory performance in sleep restriction. Based on prior findings [48, 50], we hypothesized that the exercise plus sleep restriction condition would show better memory performance than the sleep restriction only condition and comparably to the average sleep condition, and that SWS percentage would statistically mediate the difference between the exercise plus sleep restriction and average sleep conditions.

## Materials and methods

### Participants

Ninety-two young adults (49 females, 43 males; age range 18–35 years) were recruited between November 2022 and November 2024 from Concordia University, the surrounding community, and social media platforms (e.g. Facebook). Participants were included if they were healthy, good sleepers between 18 and 35 years old, and were of varying physical activity levels, quantified by metabolic equivalent (MET) minutes per week [55]. We excluded participants who had current or past sleep, psychological, neurological, or other medical conditions. Participants who were shift workers or had traveled across more than one time zone within the month preceding participation were excluded. We also excluded participants who were taking medications known to affect sleep or cognition, as well as those with uncorrected visual problems. In addition, individuals with obesity (BMI ≥30 kg/m^2^) were excluded due to potential associations with obstructive sleep apnea and cognitive impairment [56]. All participants underwent baseline PSG to objectively rule out sleep disorders before inclusion. The study protocol was approved by the Concordia University Institutional Review Board (certificate #: 30012459), and all participants provided written informed consent.

### Study design and overview of study procedures

This study employed a laboratory-based, between-subjects design with four experimental groups, defined by sleep opportunity and exercise condition: (1) **S**_**8**_: average sleep opportunity only (8.5 h), (2) **S**_**5**_: restricted sleep opportunity only (4.5 h), (3) **ExS**_**5**_: exercise before restricted sleep, and (4) **ExS**_**8**_: exercise before average sleep opportunity. Participants visited the Sleep Laboratory at Concordia University for a baseline night and an experimental overnight session a week later (Figure 1).

**Figure 1:**
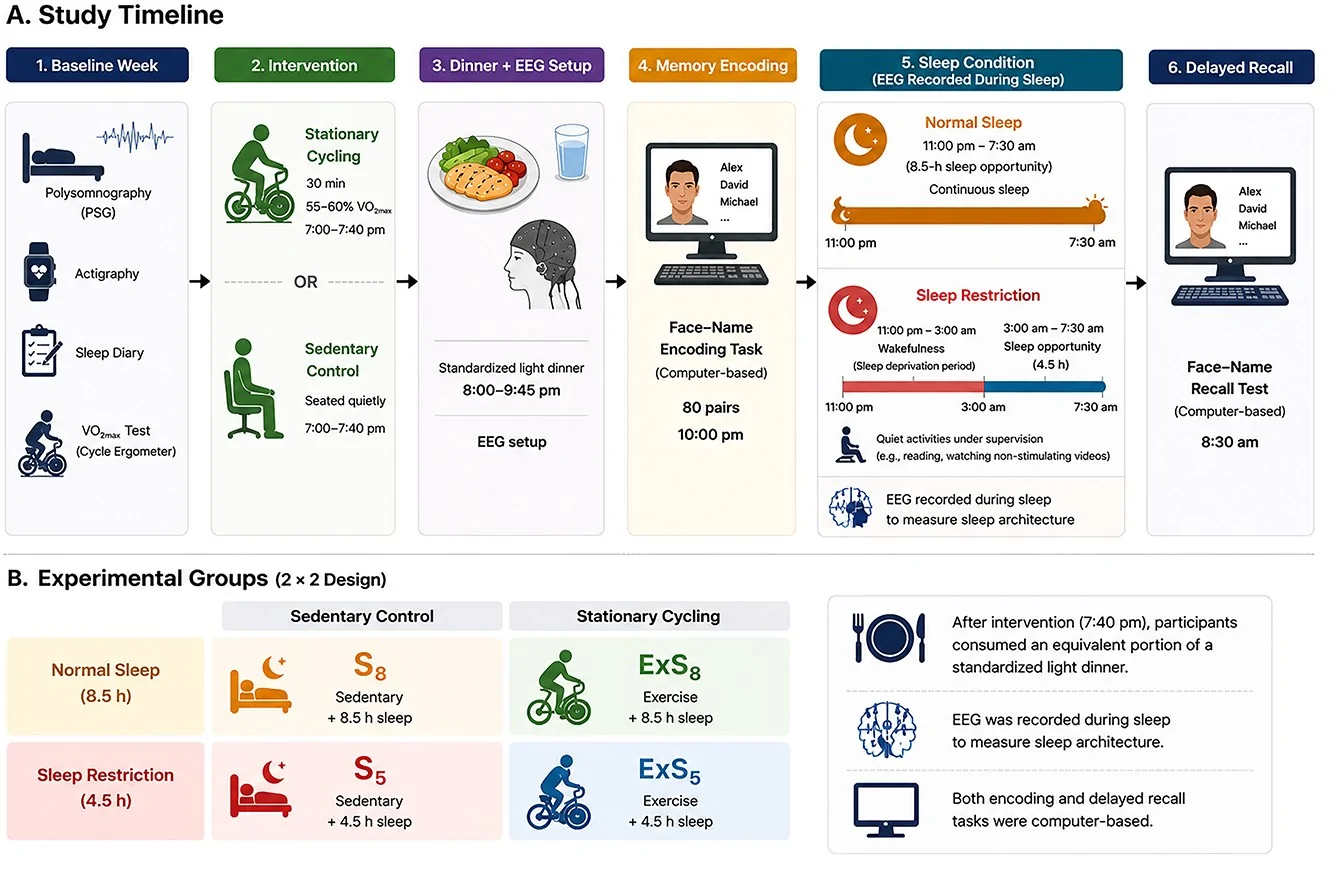
Study protocol. PSG: polysomnography, EEG: electroencephalography, VO_2max:_ maximal oxygen consumption/peak oxygen uptake. S_8_: average sleep only (8.5 h/night), S_5_: restricted sleep only (4.5 h/night), ExS_5_: exercise before restricted sleep, ExS_8_: exercise before average sleep opportunity.

The participants first completed online screening questionnaires administered through Qualtrics platform to assess initial eligibility. Detailed descriptions of these questionnaires are published elsewhere [50]. Briefly, the screening questionnaires included the: (1) Pittsburgh Sleep Quality Index (PSQI) for assessing subjective sleep quality [57], (2) Epworth Sleepiness Scale (ESS) for evaluating daytime sleepiness [58], (3) Insomnia Severity Index (ISI) [59] and Ullanlinna Narcolepsy Scale (UNS) [60] for screening sleep disorders, (4) Beck Depression Inventory-II (BDI-II) [61] and the Beck Anxiety Inventory (BAI) [62] for mood or psychological status assessment, (5) Horne–Östberg Morningness–Eveningness Questionnaire (MEQ) for assessing the chronotype of participants [63], and (6) International Physical Activity Questionnaire (IPAQ, short version) for estimating the levels of physical activity of participants in MET-minutes per week. Participant eligibility was determined based on questionnaire scores meeting established cut-off values (Table S1).

### Baseline procedures

Following online screening, eligible participants completed a baseline PSG habituation night that also screened for sleep disorders. Participants in the exercise condition (ExS_5_ and ExS_8_) also completed a cardiorespiratory fitness assessment (VO_2max_) to determine the individualized moderate-intensity threshold for the exercise intervention during the experimental session. After the baseline night, participants were instructed to maintain regular sleep–wake schedules for one week prior to the experimental session. To verify compliance, participants wore an actigraph (Actiwatch Spectrum, Philips Respironics®, or ActiGraph GT3X+, ActiGraph LLC) on their non-dominant wrist and completed daily sleep diaries throughout the monitoring period (Figure 1).

### Sample size and randomization

A prior study from our laboratory [48] reported a small interaction effect between acute high-intensity evening exercise and sleep on long-term declarative memory (*partial η*^2^ = 0.033). Based on this finding, we anticipated a moderate interaction effect (partial η^2^ = 0.081) for moderate-intensity exercise. An *a-priori* power analysis was conducted using G^*^Power (version 3.1.9.6) [64] with the “ANOVA: Fixed effects, special, main effects and interaction.” Considering α = 0.05, power (1-β) = 0.80, and a 2x2 between-subjects design, a total sample of N = 92 was required to detect a moderate interaction effect (f = 0.297) between acute moderate-intensity evening exercise and sleep on long-term memory performance accuracy. Participants were randomly assigned to one of the four experimental conditions (S_8_, S_5_, ExS_5_, and ExS_8_) using computer-generated random numbers by a research assistant not involved in data collection or analysis. Group allocation remained concealed until participants’ arrival at the Sleep Laboratory on the experimental evening to minimize potential bias.

### Assessment of cardiorespiratory fitness (VO_2max_)

A progressive incremental exercise test to volitional fatigue was performed on an electrically braked stationary cycle ergometer (Lode Bike, Lode B.V. Medical Technology, Groningen, The Netherlands) to determine maximal oxygen consumption (VO₂_max_). A ramped seated cycle ergometer protocol was used, beginning with a 3-min freewheel warm-up at a cadence below 60 revolutions per minute (rpm). Thereafter, the resistance was increased by 30 W every minute, while participants maintained a cadence of 60–65 rpm, continuing until volitional exhaustion. Oxygen consumption (VO₂) was continuously measured on a breath-by-breath basis using a metabolic cart (Ultima™ CardiO₂®, MGC Diagnostics, Minnesota, United States). Participants were connected to a 12-lead electrocardiogram (ECG) for continuous monitoring of cardiac electrical activity, including heart rate (HR), and blood pressure (BP) throughout the test. The rating of perceived exertion (RPE) was recorded using the Borg 6–20 scale [65]. The test was terminated upon reaching volitional fatigue or exhaustion and/or established termination criteria [66]. Combining these parameters, a maximal test was considered to have been achieved if: (1) VO_2_ plateaued despite an increase in workload, (2) HR was ≥95% of the participant’s age-predicted maximal HR, (3) the respiratory exchange ratio (RER) was ≥1.15, and (3) RPE was >19 indicating “maximal exertion” [66]. VO₂_max_ was defined as the highest 30-s average VO₂ value, normalized to body mass. From the test, HR corresponding to the VO_2max_ and at 55%-60% of VO_2max_ were recorded.

### Experimental night procedures and outcome assessments

On the experimental day (i.e. one week after the baseline night), all participants completed an experimental night procedure that included an early evening exercise or sedentary intervention (7:00 p.m.–7:40 p.m.), memory tasks (10:00 p.m.–10:45 p.m.), and a subsequent night of either average or restricted sleep. Participants were asked to rest, hydrate and eat no less than 3 hours before arrival at the Sleep Laboratory at 6:30 p.m. Participants were instructed to refrain from caffeine, alcohol and moderate-to-vigorous physical activity for 24 hours prior to the experimental session. Participants were also asked not to take naps on the experimental day. Compliance was verified via sleep diary and/or actigraphy. After the exercise and sedentary interventions (7:40 p.m.), participants were served equivalent portions of a standardized light dinner from the Nutrition Suite of the School of Health or frozen dinners provided by the research team. Except for water ad libitum, participants were instructed to refrain from taking coffee, caffeine and any other beverages before bedtime and the next morning before the delayed retrieval task. No additional food was permitted until after the delayed retrieval. Following the standardized dinner, participants underwent PSG setup for overnight sleep recording (8:00 p.m.–9:45 p.m.). At 10:00 p.m., participants completed the encoding session of the memory task—a learning session consisting of 80 face-name pairs, followed by an immediate recall at 10:15 p.m. The immediate retrieval task included 120 face-name pairs (80 studied and 40 novel faces).

Participants then had either an average sleep opportunity (8.5 hours/nigh; 11:00 p.m.–7:30 a.m.) or a restricted sleep opportunity (4.5 hours/night; 3:00 a.m.–7:30 a.m.), both verified using PSG. Following sleep, participants performed a delayed retrieval task comprising the same number of face-name pairs as the immediate retrieval in the morning at 8:30 a.m. (Figure 1).

### Moderate-intensity exercise intervention and sedentary protocol

The timing and intensity of the exercise intervention were selected in accordance with previous studies [48, 50, 67], ensuring that exercise occurred prior to the learning task and more than 3 hours before bedtime [23, 68]. Participants in the ExS_5_ and ExS_8_ groups performed a moderate-intensity cardiovascular exercise session on a cycle ergometer at 7:00 p.m. The exercise session began with a 5-minute warm-up at an RPE of 6–10, followed by 30 minutes of continuous cycling at a target HR corresponding to 55%–60% of VO₂_max_ (i.e. 123–130 bpm), as determined from each participant’s graded exercise test. The session ended with a 5-minute low-intensity cycling and stretching cool-down (RPE: 6–9). To objectively verify adherence to the prescribed intensity, participants wore a Polar heart rate monitor, and HR was continuously monitored throughout the session. Blood pressure was measured before and after the exercise session, whereas RPE was recorded before, during, and after exercise to monitor subjective effort levels. The exercise session was supervised by an exercise physiologist. In contrast, participants in the sedentary groups (S_5_ and S_8_) followed a 40-minute seated rest protocol during the same period (7:00–7:40 p.m.) to control for time and environmental exposure. They sat quietly in a comfortable chair for 40 minutes and were permitted to read, use a laptop or tablet, or listen to music, but were asked to avoid physical activity during the sedentary intervention period.

### Sleep restriction protocol

Participants assigned to the average sleep conditions (S_8_ and ExS_8_) went to bed shortly after completing the memory tasks and maintained a standard sleep schedule (11:00 p.m.–7:30 a.m.). In contrast, those in the S_5_ and ExS_5_ conditions remained awake until 3:00 a.m., after which they were allowed a 4.5-hour sleep opportunity (3:00 a.m.–7:30 a.m.). During the sleep deprivation period (11:00 p.m.–3:00 a.m.), sleep-restricted participants were required to remain sedentary in the Sleep Laboratory but were occasionally permitted to stand, stretch, or take brief (<2 minutes) walks within the laboratory. Participants were allowed to engage in quiet activities such as Internet browsing, reading, studying, or watching movies. Participants were allowed to use personal devices (laptops, tablets, and smartphones) but were instructed to use blue-light filters (e.g. night mode). Screen time was not restricted but all groups were treated identically. All participants refrained from consumption of coffee, caffeinated beverages, or any fluids other than water. Wakefulness was continuously monitored by research assistants, who also engaged participants in light conversation to minimize drowsiness. Throughout the sleep deprivation period, the laboratory and bedrooms were kept at the same ambient temperature and light levels as during the daytime.

### Assessment of primary and secondary outcomes

#### Long-term declarative memory assessed with the discriminability index (d′)

A modified version of the face–name association task [69] was employed to assess long-term declarative memory. The task comprised three stages: encoding (learning), immediate retrieval, and delayed retrieval. During encoding, participants viewed 80 face–name pairs, each shown once on a computer screen for 5 seconds using PsychoPy software (v3.0.7) and were instructed to memorize them for subsequent retrievals. The encoding phase lasted approximately 10 minutes.

For both retrieval sessions, participants were presented with 120 faces consisting of the 80 previously studied face–name pairs in random order and 40 novel faces (foils), which were different for the immediate and delayed retrieval tasks. Participants were instructed to identify whether each face was previously studied (old) and, if so, select the corresponding name, or to indicate “new” if the face was unfamiliar. The immediate retrieval occurred approximately 5–10 minutes after encoding, whereas the delayed retrieval was conducted the following morning after either the average or restricted sleep opportunity. Each retrieval session lasted approximately 20 minutes. Upon completion, participants rated their subjective sleepiness using the 9-item Karolinska Sleepiness Scale (KSS), where 1 indicates “extremely alert” and 9 indicates “very sleepy.”

The ***primary** outcome* measure was long-term memory performance, measured using the discriminability index (*d′*) at delayed retrieval. The *d′* reflects a participant’s ability to discriminate between previously studied and novel stimuli [70] and was calculated using the formula: ${d}^{\prime }=\mathrm{zHT}-\mathrm{zFA}$ [70, 71]; where the Z_HT_ and Z_FA_ are the *z* scores of the hit (HT) and the false alarm (FA) rates, respectively. The HT represents the probability of correctly classifying old stimuli as old, whereas the FA represents the probability of incorrectly classifying new stimuli as old. Higher *d′* values indicate greater sensitivity to distinguish targets from foils [70].

***Secondary outcomes*** included the difference in *d′* between delayed and immediate retrievals, immediate and delayed face-name retrieval accuracy (i.e. proportion of studied old stimuli correctly recalled), reaction time (RT) during retrieval, and self-reported ratings of motivation, perceived task difficulty, performance, and sleepiness. Motivation, task difficulty, and performance were each rated on a 0–10 scale (low to high), and sleepiness was assessed using the KSS at the end of each retrieval session.

#### Sleep architecture

Participants completed two overnight PSG sessions: a baseline and an experimental night. The PSG protocols were identical except that the experimental recording included only EEG, electrooculography (EOG), submental electromyography (EMG), and ECG. EEG signals were recorded with a 0.3–100 Hz bandpass filter and a 512-Hz sampling rate, referenced to the mastoids. Electrode placement followed the international 10–20 system and included frontal (F_3_/F_4_), central (C_3_/C_4_), parietal (P_3_/P_4_), occipital (O_1_/O_2_), and midline (F_pz_, F_z_, C_z_, P_z_) sites. Pre-gelled ECG electrodes were applied to the chest and rib areas in a Lead II configuration and recorded at 512 Hz using Domino software (version 2.8.0). Audiovisual recordings were monitored throughout the night by trained sleep technicians and research assistants. Sleep stages were scored manually in 30-second epochs according to the standard criteria of the American Academy of Sleep Medicine (AASM) [72] by two independent scorers (EF and MM) who were unblinded to group allocations. EEG data quality was assessed visually before sleep scoring and no participant had any channels excluded due to artifacts.

Sleep variables included total sleep time (TST), sleep efficiency (ratio of TST to time in bed after lights off), wake after sleep onset (WASO: total wake time after sleep onset), sleep onset latency (SOL: time to fall asleep after lights off), number of awakenings, and the percentage and duration of REM sleep and non-REM (NREM) sleep stages N1, N2, and N3 (SWS). The percentage of TST spent in SWS (SWS%) was the primary mediator (pre-plan), as it provides a more sensitive and robust indicator of sleep quality in response to acute exercise than other sleep stages [73].

### Statistical analysis

Data were analyzed using SPSS (version 30). For all analysis of variance (ANOVA) models, normality of residuals was verified using Shapiro–Wilk tests (*p* > .05). Homogeneity of variance was confirmed using Levene’s test (*p* > .05, for all comparisons). A 2 × 2 ANOVA was conducted to examine the main effects of exercise and sleep conditions, as well as their interaction on memory performance (*d′*) at delayed retrieval (primary outcome), immediate retrieval, and their difference scores (secondary outcomes), with sex and chronotype (MEQ score) included as covariates. In addition, the 2 × 2 ANOVA with interaction analysis was repeated for immediate and delayed retrieval face–name accuracy scores as secondary outcomes. When significant interactions were observed, Bonferroni-adjusted post hoc tests were performed to determine group differences in *d′* and face-name accuracy scores. Analysis of covariance (ANCOVA) was conducted to examine the effects of immediate retrieval *d′* and face-name accuracy scores on delayed retrieval *d′* and delayed face-name retrieval, respectively. Immediate retrieval *d′* and face-name accuracy score were included in each model as covariate in addition to sex and MEQ scores. One-way ANOVA was used to compute group differences in sleep variables, RT, KSS scores, and ratings of motivation and performance during retrievals. A 2×2 ANOVA was also used to verify group differences in the sleep variables.

A mediation analyses was conducted using Model 4 of Hayes’ PROCESS macro (version 4.2) implemented in SPSS [74], with experimental condition as the independent variable, SWS percentage (SWS%) as the mediator, and delayed retrieval *d′* as the dependent variable, while MEQ score was included as a covariate. The mediator variable (SWS%) was z-standardized before mediation. An indirect (mediation) effect was estimated using percentile bootstrap confidence intervals based on 5000 bootstrap samples (95% confidence level). Statistical significance of the mediation effect was determined by whether the confidence interval excluded zero. Exploratory mediation analyses were conducted for the other sleep stages. Additionally, exploratory moderation analyses were also conducted to examine the moderating effects of sleep architecture on the relationship between experimental condition and long-term memory using Model 1 of the PROCESS macro. A two-tailed statistical significance was set at *p* < .05.

## Results

### Participant characteristics

Eighty-eight participants (mean ± SD: age = 23.0 ± 3.8 years, body mass index [BMI] = 23.4 ± 2.9 kg/m^2^, 52% female) were included in the final analysis. Four participants from the initial cohort (*N* = 92) were excluded from the analysis due to withdrew from study (*n* = 3) or non-adherence to the experimental protocol (*n* = 1; Figure 2). On average, participants reported moderate-to-vigorous levels of physical activity (IPAQ; 3975.32 ± 3346.51 metabolic equivalent [MET]-min per week), and an intermediate chronotype (MEQ; 42.60 ± 11.60; Table 1). Groups did not differ significantly in demographics, subjective sleep quality (PSQI), psychological measures (BDI-II and BAI), or fitness (exercise groups only; *ps* > 0.05). However, a significant difference in MEQ scores was observed between the ExS_8_ and S_5_ (*p* = .036), with participants in the exercise conditions (ExS_5_ and ExS_8_) reporting a more evening chronotype compared with the sedentary conditions (S_5_ and S_8_). The sample was racially and ethnically diverse, comprising approximately 62% White, 24% Asian, 9% Latino, and 5% Black participants (Table 1). No significant differences were found between the ExS_5_ and ExS_8_ groups in VO_2max_ or maximal heart rate (HR_max_; *ps* > 0.05; Table 1).

**Figure 2:**
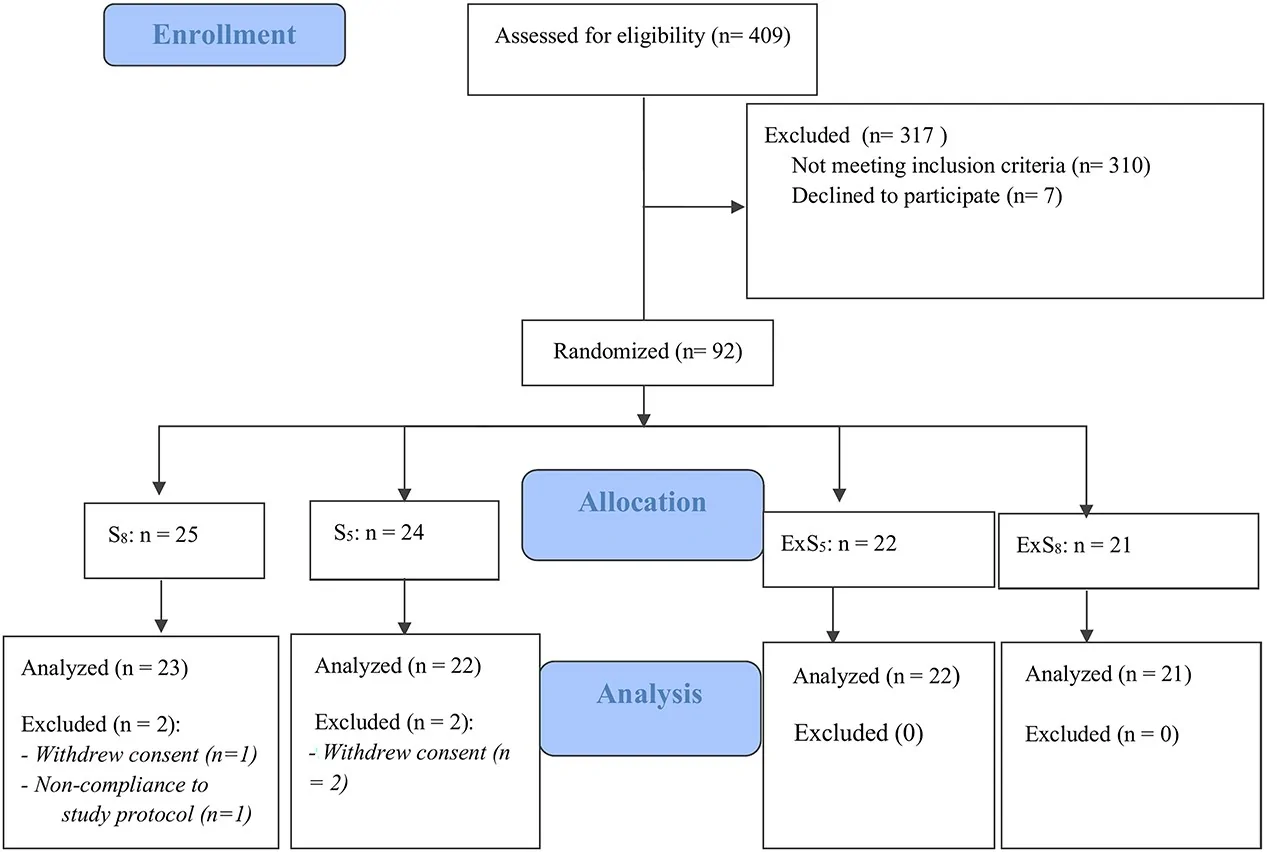
Flow diagram showing participant recruitment. S_8_: average sleep only (8.5 h/night), S_5_: restricted sleep only (4.5 h/night), ExS_5_: exercise before restricted sleep, ExS_8_: exercise before average sleep opportunity.

**Table 1:** Participant characteristics

Variable	Total (*N* = 88)	S_8_ (*n* = 23)	S_5_ (*n* = 22)	ExS_5_ (*n* = 22)	ExS_8_ (*n* = 21)
Sex, f(M)	46 (42)	14 (9)	12 (9)	19 (13)	10 (11)
Age (years)	24.0 ± 3.8	24.0 ± 3.5	22.9 ± 3.3	23.7 ± 4.3	25.6 ± 3.8
BMI (kg/m^2^)	23.4 ± 3.3	23.4 ± 3.4	23.1 ± 3.6	23.6 ± 3.0	23.5 ± 3.4
PSQI	3.5 ± 1.2	3.2 ± 1.3	3.6 ± 1.2	3.6 ± 1.0	3.8 ± 1.1
ESS	5.2 ± 3.2	5.2 ± 2.8	5.7 ± 3.8	5.0 ± 3.0	5.0 ± 3.3
MEQ	42.6 ± 11.6	45.6 ± 11.1	46.5 ± 9.1	41.1 ± 14.1	36.7 ± 9.5^*^
BDI	4.1 ± 4.6	4.0 ± 4.8	4.9 ± 5.1	3.6 ± 5.4	3.7 ± 3.1
BAI	5.5 ± 5.5	5.8 ± 5.9	6.9 ± 6.6	5.3 ± 5.7	4.0 ± 2.7
ISI	3.1 ± 2.5	2.5 ± 2.6	3.4 ± 3.1	2.9 ± 2.2	3.7 ± 2.3
UNS	7.1 ± 3.7	7.2 ± 5.1	6.9 ± 3.5	6.8 ± 3.6	7.3 ± 2.3
IPAQ (MET-min/wk)	3975.3 ± 3346.5	4099.2 ± 3233.5	3152.6 ± 1947.4	4468.7 ± 5016.2	4203.5 ± 2566.1
VO_2max_ (ml/min/kg)	-	-	-	36.8 ± 11.4	37.0 ± 11.0
HR_max_ (bpm)	-	-	-	172.7 ± 12.9	174.0 ± 10.9
RPE	-	-	-	18.2 ± 1.3	17.3 ± 1.2

*N* = 88. Sex is presented as numbers; all other data are presented as means ± SD.

S_8_: average sleep only, S_5_: restricted sleep only, ExS_5_: exercise before restricted sleep, ExS_8_: exercise before average sleep opportunity. F: females (52%), M: males (48%), PSQI: Pittsburgh Sleep Quality Index, ESS: Epworth Sleepiness Scale, MEQ: Morningness-Eveningness Questionnaire, BDI: Beck Depression Inventory, BAI: Beck Anxiety Inventory, ISI: Insomnia Severity Index, UNS: Ullanlinna Narcolepsy Scale, IPAQ: International Physical Activity Questionnaire, BMI: Body Mass Index, MET-min/wk: Metabolic Equivalent-Minutes per week, VO_2max_: maximal oxygen consumption, HR_max_: maximal heart rate, RPE: rating of perceived exertion.

^*^ *MEQ score of* S_5_ vs. ExS_8_, *p* = .036.

### Memory performance across the groups

Moderate exercise significantly interacted with sleep condition to influence delayed retrieval performance (*d′*; Table 2). There was a significant interaction effect (*F*(1, 79) = 4.703, *p* = .033, *ηp^2^* = 0.056) between the exercise [*F*(1, 79) = 1.051, *p* = .308, *ηp^2^* = 0.013)] and sleep [*F*(1, 79) = 6.881, *p* = .010, *ηp^2^* = 0.080)] conditions on delayed retrieval *d′*, after controlling for sex and chronotype (MEQ score).

**Table 2:** 2 × 2 ANOVA with interaction analysis between the exercise and sleep conditions on long-term memory performance (Delayed Retrieval *d′*) with sex and chronotype (MEQ score) as covariates

Variables	Source	*SS*	*df*	*MS*	*F*	*P*	*η* _*p*_ ^*2*^
Delayed retrieval *d′*	Sex	0.069	1	0.069	0.047	.829	0.001
	MEQ score	1.776	1	1.776	1.204	.276	0.015
	Exercise conditions	1.550	1	1.550	1.051	.308	0.013
	Sleep conditions	10.148	1	10.148	6.881	.010^*^	0.080
	Exercise x sleep interaction	6.936	1	6.936	4.703	.033^*^	0.056
	Error	116.513	79	1.475			
	Total	136.538	85				
Immediate retrieval *d′*	Sex	0.099	1	0.099	0.060	.807	0.001
	MEQ score	2.967	1	2.967	1.800	.184	0.022
	Exercise conditions	0.390	1	0.390	0.237	.628	0.003
	Sleep conditions	0.952	1	0.952	0.578	.449	0.007
	Exercise x sleep interaction	0.446	1	0.446	0.270	.604	0.003
	Error	130.184	79	1.648			
	Total	136.812	85				
	Total	136.538	85				
Difference in *d′* (delayed – immediate r)	Sex	1.697	1	1.697	1.607	.209	0.020
	MEQ score	0.675	1	0.675	0.639	.426	0.008
	Exercise conditions	12.864	1	12.864	12.182	<.001^*^	0.134
	Sleep conditions	12.857	1	12.857	12.176	<.001^*^	0.134
	Exercise x sleep interaction	6.938	1	6.938	6.570	.012^*^	0.077
	Error	83.417	79	1.056			
	Total	124.031	85				

*N = 88*. Exercise conditions (exercise vs. sedentary, *n* = 43), sleep conditions (average vs. restricted sleep, *n* = 45). S_8_: average sleep only (n = 23), S_5_: restricted sleep only (n = 22), ExS_5_: exercise before restricted sleep (n = 22), ExS_8_: exercise before average sleep opportunity (n = 21). *d′*: discriminability index, MEQ: Morningness-Eveningness Questionnaire, *η_p_^2^*: partial eta-squared, *SS*: Type III Sum of Squares, *df*: degree of freedom, *MS*: Mean Square.

*P-values* are Bonferroni-adjusted.

^*^ *p* < .05.

Post hoc comparisons with Bonferroni adjustment showed that the delayed retrieval *d′* of the ExS_5_ group (mean ± SD: 0.07 ± 0.89) did not differ significantly from the S_8_ group (0.58 ± 1.20, *t*(79) = 1.10, *p* = .274, Cohen’s d = 0.25) or ExS_8_ (0.21 ± 1.30, *t*(79) = 0.32, *p* = .747, Cohen’s d = 0.04). However, the ExS_5_ group performed significantly better than the S_5_ group (-0.60 ± 1.38, *t*(79) = 2.25, *p* = .028, Cohen’s *d* = -0.51). The S_5_ group also showed significantly lower *d′* score compared with both the S_8_ (*t*(79) = 3.47, *p* < .001, Cohen’s d = -0.78) and ExS_8_ (*t*(79) = 2.53, *p* = .013, Cohen’s d = -0.57) groups (Figure 3). In addition, the *d′* of ExS_8_ (0.21 ± 1.30) did not differ significantly from that of the S_8_ group (0.58 ± 1.20, *t*(79) = 0.73, *p* = .467, Cohen’s d = 0.08).

**Figure 3:**
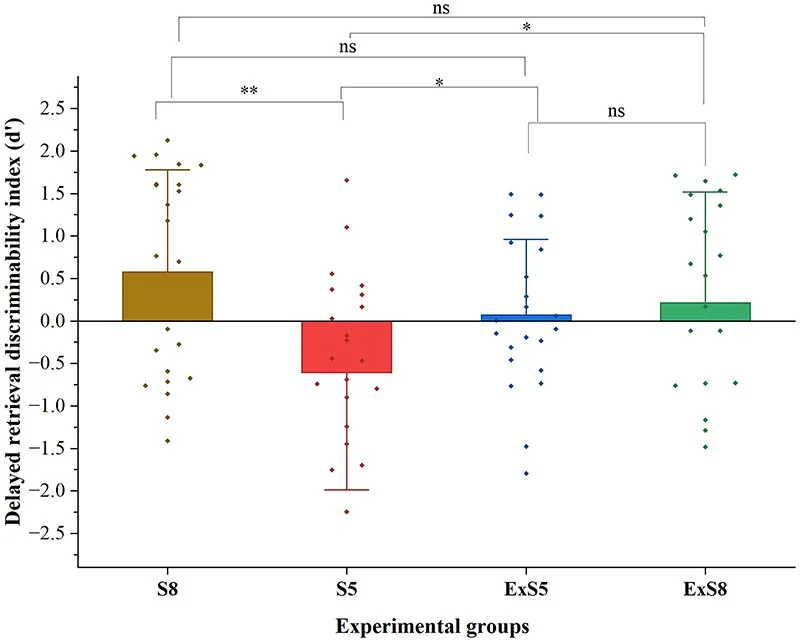
Comparisons of long-term memory performance (delayed retrieval *d′*) between the experimental groups. S_8_: average sleep only (8.5 h/night, *n* = 23), S_5_: restricted sleep only (4.5 h/night, *n* = 22), ExS_5_: exercise before restricted sleep (*n* = 22), ExS_8_: exercise before average sleep opportunity (*n* = 21). Means ± SD. Statistical test: 2×2 analysis of variance with interaction and *P*-values of post hoc tests were Bonferroni-adjusted for multiple comparisons. **Ns**: not significant, *p* > .05. ^*^p < .05; ^**^p < .001. ♦diamonds indicate the data points.

We found no statistically significant interaction effect between the exercise (*F*(1, 85) = 0.237, *p* = .628, *η_p_^2^* = 0.003) and sleep (*F*(1, 79) = 0.578, *p* = .449, *η_p_^2^* = 0.007) conditions on the *d′* at the immediate retrieval (*F*(1, 79) = 0.270, *p* = .604, *ηp^2^* = 0.003). This suggests that any group differences in encoding, if present, were not large enough to detect in this sample (Table 2; Figure S1).

We found significant main effects of exercise (*F*(1, 79) = 12.864, *p* < .001, *ηp^2^* = 0.134) and sleep (*F*(1, 79) = 12.857, *p* < .001, *ηp^2^* = 0.134), and a significant interaction effect (*F*(1, 79) = 6.570, *p* = .012, *ηp^2^* = 0.077) for the difference in *d′* after controlling for sex and chronotype (Table 2, Figure S2). Post hoc comparisons with Bonferroni adjustment revealed that the ExS_5_ group (mean ± SD: 0.36 ± 0.8) did not differ significantly from the S_8_ (0.23 ± 1.37, *t*(79) = 0.15, *p* = .881, Cohen’s d = 0.034) or ExS_8_ (0.63 ± 0.68, *t*(79) = 0.65, *p* = .516, Cohen’s d = 0.15), but showed a significantly greater difference than the S_5_ (-1.13 ± 1.02, *t*(79) = 4.33, *p* < .001, Cohen’s *d* = -0.97). The S_5_ group also showed a significantly lower *d′* difference score compared with the S_8_ (*t*(79) = 4.38, SE = 0.310, *p* < .001, Cohen’s d = -0.99) and ExS_8_ (*t*(79) = 4.91, *p* < .001, Cohen’s d = -1.105; Table 2, Figure S2). These results indicate that the exercise intervention was associated with improved sleep-dependent long-term memory consolidation in the ExS_5_ group compared to the S_8_ and enhanced memory consolidation relative to the S_5_ condition. However, the *d′* difference score of the ExS_8_ (0.63 ± 0.68) did not differ significantly from that of the S_8_ group (0.23 ± 1.37, *t*(79) = 0.79, *p* = .433, Cohen’s d = 0.18).

A confirmatory 2 × 2 ANCOVA on delayed retrieval *d′* revealed that the exercise (*F*(1, 78) = 4.372, *p* = .040, *η_p_^2^* = 0.053)) x sleep (*F*(1, 78) = 22.969, *p* < .001, *η_p_^2^* = 0.227)) interaction remained statistically significant, even after controlling for immediate *d′* in addition to sex and MEQ score as covariates (*F*(1, 78) = 7.142, *p* = .009, *ηp^2^* = 0.084), consistent with the primary analysis (Table S2). Similarly, there was a significant exercise (*F*(1, 78) = 16.948, *p* < .001, *ηp^2^* = 0.179) x sleep (*F*(1, 78) = 23.752, *p* < .001, *ηp^2^* = 0.233) interaction on delayed retrieval face-name accuracy after controlling for immediate retrieval face-name accuracy, sex and MEG score (*F*(1, 78) = 23.512, *p* < 0.001, *η_p_^2^* = 0.232; Table S3; Figure 4). Bonferroni post hoc comparisons are reported in Table S4. There was no exercise x sleep interaction for immediate face-name retrieval accuracy (Figure S3).

**Figure 4:**
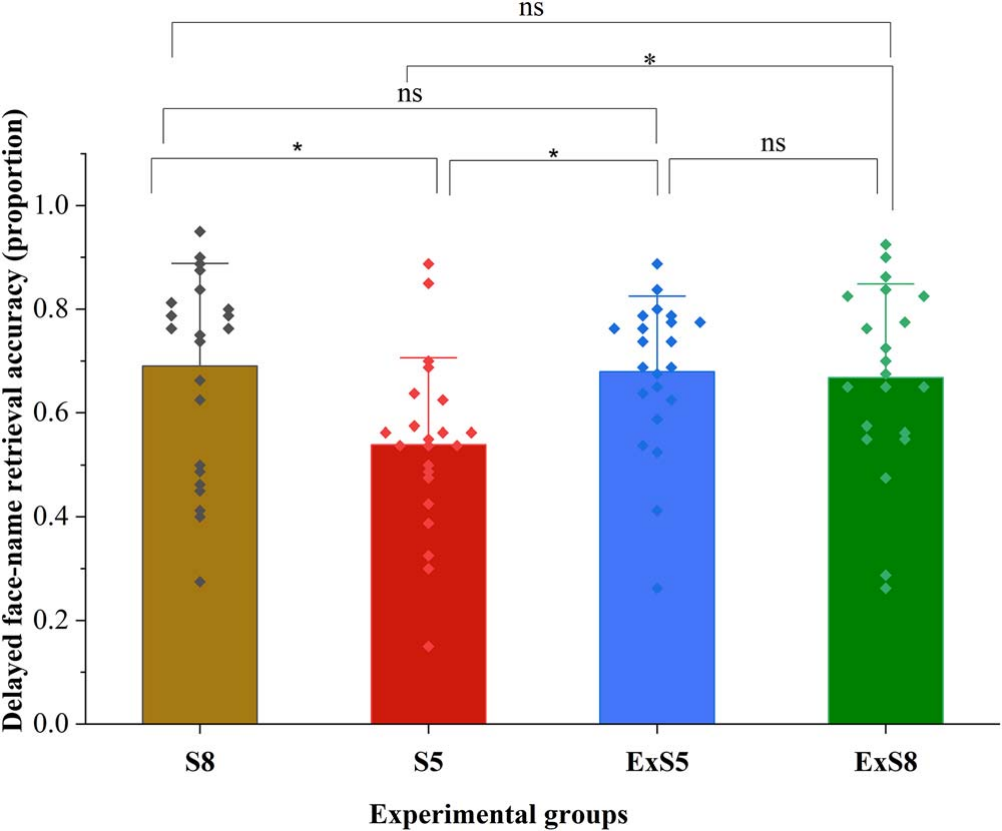
Comparisons of delayed face-name retrieval accuracy between experimental groups. S_8_: average sleep only (8.5 h/night, *n* = 23), S_5_: restricted sleep only (4.5 h/night, *n* = 22), ExS_5_: exercise before restricted sleep (*n* = 22), ExS_8_: exercise before average sleep opportunity (*n* = 21). Means ± SD. Statistical test: 2X2 analysis of variance with interaction and *P*-values of post hoc tests were Bonferroni-adjusted for multiple comparisons. **Ns**: not significant, *p* > .05. ^*^p < .05; ^**^p < .001. ^♦^diamonds indicate the data points.

Additional secondary analyses examined participants’ subjective ratings of motivation, perceived task difficulty, and perceived performance (all assessed on a visual analogue scale; 0 = low, 10 = high), as well as sleepiness ([KSS]; 1 = extremely alert, 9 = very sleepy) during the retrieval tasks. A one-way ANOVA revealed no significant group differences in motivation (*p* = .251), performance (*p* = .079), or task difficulty (*p* = .265) during the immediate retrieval task, but a significant effect for KSS score (*p* = .018) (Figure S4, A–E). Post hoc comparisons showed that the ExS_5_ (mean ± SD: 4.77 ± 2.4) reported significantly lower KSS score compared to the ExS_8_ group (6.57 ± 1.33, *p* = .019) at the immediate retrieval (Figure S4, A–E). During delayed retrieval, no significant group differences were found for motivation (*p* = .380), perceived performance (*p* = .194), or task difficulty (*p* = .431), but sleepiness differed significantly (*p* < .001) (Figure S5, A–E). The S_5_ group (5.18 ± 2.28) reported higher KSS score than the S_8_ (3.65 ± 1.70, *p* < .001) and ExS_8_ (3.76 ± 1.48, *p* < .001) groups. The ExS_5_ also reported significantly higher KSS score relative to the S_8_ (*p* = .039) but did not differ significantly from ExS_8_ group (*p* = .079). Reaction times during both immediate and delayed retrievals did not differ significantly across the groups (*ps* > 0.05) (Figure S4E & Figure S5E). These results suggest that while motivation, perceived performance, and task difficulty did not vary across groups, subjective sleepiness reflected the sleep conditions, supporting protocol compliance.

### Comparisons of sleep architecture between the groups

No significant group differences were found in baseline PSG sleep variables one week prior to the experimental session (*p* > .05; Table S5). Comparisons of sleep parameters during the experimental night are summarized in Table 3. Expected differences between sleep-restricted and full-sleep groups were observed for TIB and TST. Sleep onset latency differed significantly across the groups (*F*(3, 84) = 7.673, *p* < .001, *η^2^* = 0.215). Bonferroni post hoc comparisons revealed that SOL was significantly shorter in the S_5_ than in the S_8_ group (*t*(84) = 4.39, *p* < .001, Cohen’s d = 0.96) and in the ExS_5_ compared with the S_8_ (*t*(84) = 3.79, *p* = .002; Cohen’s d = 0.83). Significant group differences were observed in both the percentage of TST spent in SWS (*F*(3, 84) = 5.724, *p* = .001, *η^2^* = 0.170) and the absolute duration of SWS (*F*(3, 84) = 15.152, *p* < .001, *η^2^* = 0.351; Table 3). Specifically, the proportion of TST spent in SWS was significantly higher in the ExS_5_ than in the S_8 (*t*(84) = 3.54,_  *p* = .004, Cohen’s d = 0.772) and higher in the S_5_ than in the S_8 (*t*(84) = 3.04,_  *p* = .019, Cohen’s d = 0.662). SWS percentage did not differ significantly between S_5 and_ ExS_5_ (*t*(84) = 0.50, *p* = .999, Cohen’s d = 0.109), and also between the ExS_5_ and ExS_8 (*t*(84) = 1.80,_  *p* = .064, Cohen’s d = 0.393; Table 3).

**Table 3:** Comparisons of Experiment Night Sleep Architecture between the Groups

Variable	S _8_ (*n* = 23) Mean ± SD	S _5_ (*n* = 22) Mean ± SD	ExS _5_ (*n* = 22) Mean ± SD	ExS _8_ (*n* = 21) Mean ± SD	*P* ^γ^
TIB (h)^#^	8.4 ± 0.4	4.5 ± 0.1	4.5 ± 0.2	8.3 ± 0.3	<.001^*^
TST (h)^#^	7.6 ± 0.5	4.2 ± 0.2	4.2 ± 0.3	7.7 ± 0.6	<.001^*^
SOL (min)^#^	19.7 ± 17.3	6.0 ± 4.0	7.9 ± 6.0	12.5 ± 8.6	<.001^*^
AWK (number)	15.9 ± 11.1	7.7 ± 4.9	6.2 ± 4.1	12.1 ± 8.6	<.001^*^
WASO (min)	48.6 ± 35.2	16.5 ± 9.9	20.8 ± 24.9	33.4 ± 29.4	<.001^*^
SE (%)	90.5 ± 6.4	94.3 ± 3.1	94.1 ± 3.5	93.3 ± 5.9	.042^*^
N1 (% of TST)	5.3 ± 3.6	3.2 ± 3.5	3.0 ± 2.7	4.2 ± 3.5	.092
N1 (min)	23.6 ± 15.7	7.8 ± 8.4	7.5 ± 6.9	19.1 ± 16.5	<.001^*^
N2 (% of TST)	47.8 ± 8.6	42.9 ± 6.5	42.5 ± 7.6	42.5 ± 7.9	.079
N2 (min)^#^	216.0 ± 34.7	108.4 ± 16.5	107.6 ± 19.7	195.9 ± 36.8	<.001^*^
SWS (% of TST)^#^	27.7 ± 6.2	34.8 ± 7.3	35.9 ± 9.2	29.7 ± 8.4	<.001^*^
SWS (min)#	127.2 ± 34.5	87.8 ± 18.8	90.8 ± 22.3	136.9 ± 39.9	<.001^*^
REM (% of TST)^#^	19.5 ± 5.0	19.1 ± 6.4	18.6 ± 5.2	23.6 ± 6.3	.020^*^
REM (min)^#^	89.5 ± 24.7	48.7 ± 17.4	47.3 ± 14.0	109.9 ± 32.9	<.001^*^
REM Lat (min)	84.4 ± 22.8	73.1 ± 25.7	70.8 ± 18.9	81.9 ± 26.4	.162

*N* = 88. S_8_: average sleep only, S_5_: restricted sleep only, ExS_5_: exercise before restricted sleep, ExS_8_: exercise before average sleep opportunity. SD: standard deviation, TIB: time in bed, TST: total sleep time, AWK: number of awakenings after sleep onset, SOL: sleep onset latency, SE: sleep efficiency, WASO: wake after sleep onset, NREM: percentage and duration of non-rapid eye movement sleep stages N1 and N2, SWS: slow-wave sleep, REM: rapid eye movement sleep.

γ *P-values* are based on One-way ANOVA with Bonferroni correction for multiple comparisons.

# Results consistent with 2 x2 ANOVA with interaction effects, *p* < .05.

^^*^^ *p* < .05.

### Mediation of memory effects of acute exercise by the percentage of slow-wave sleep

The SWS percentage statistically mediated the effect of exercise on delayed retrieval *d′* (ExS_5_ vs S_8_). The experimental condition (ExS_5_ vs. S_8_) significantly predicted SWS percentage (*B* = 0.490, *SE* = 0.138, *p* = .001), which, in turn, significantly predicted delayed retrieval *d′* (*B* = 0.214, *SE* = 0.174, *p* = .014; Figure 5). The total effect (*c*) of the model was statistically significant (-0.253, *SE* = 0.158, *p* = .012), whereas the direct effect (c*′*) was not (-0.358, *SE* = 0.179, *p* = .052). The 95% bootstrap confidence interval (CI) for the indirect effect (*ab*) did not include zero (*B* = 0.105, *SE* = 0.091, 95% CI (0.073, 0.924)), indicating a statistically significant mediation of the exercise effect on delayed retrieval *d′* through the percentage of SWS in the ExS_5_ vs. S_8_ condition (Figure 5).

**Figure 5:**
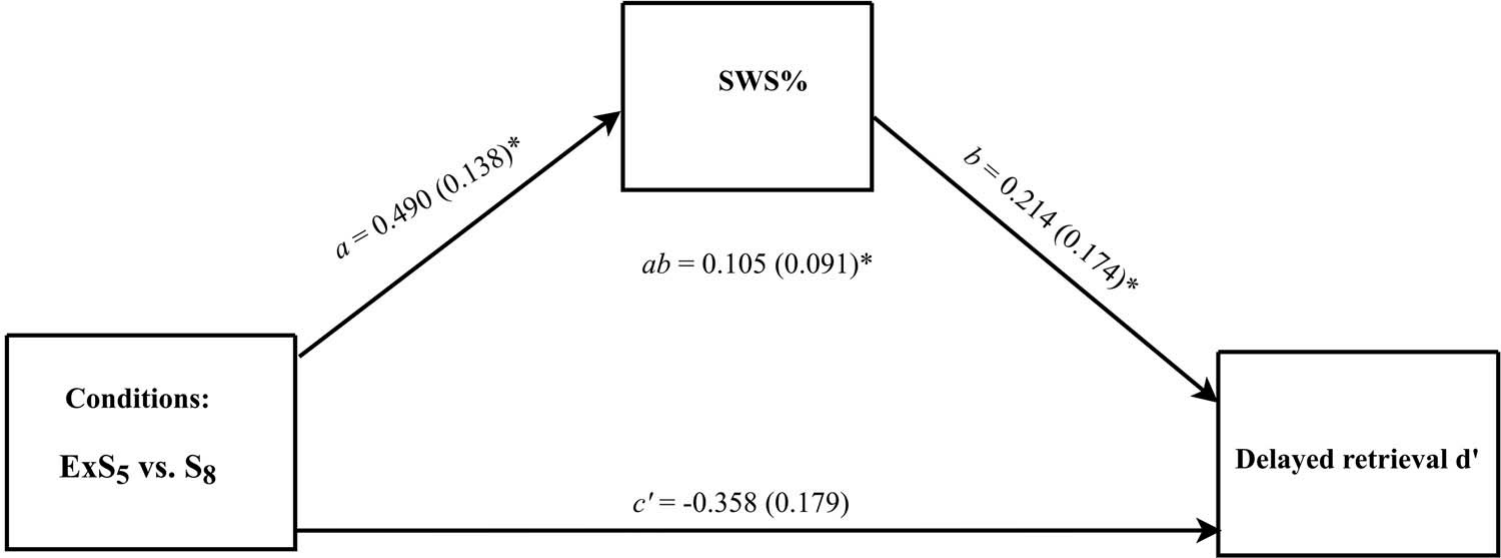
Path diagram showing the mediation of the effects of experimental condition (ExS_5_ vs. S_8_) on delayed retrieval d′ through the percentage of slow-wave sleep. S_8_: average sleep only (*n* = 23), ExS_5_: exercise before restricted sleep (*n* = 22), SWS%: percentage of sleep time spent in slow-wave sleep, ***d**′*: discriminability index at delayed retrieval, ***a***: mediation model coefficient for path ***a*** (conditions predicting SWS%), ***b***: mediation model coefficient for path ***b*** (conditions and SWS% predicting *d′*), *SE*: standard error, ***c′***: direct effect of the conditions on *d′* independent of the effects of mediators. Data are path coefficients (SE). Indirect or mediation effect **(*ab*)** 95% bootstrap confidence interval (*B* = 0.105, *SE* = 0.091, 95% CI (0.073, 0.924)). ^*^p < .05.

Exploratory analyses additionally revealed that stage N2 sleep duration (minutes) statistically mediated the effect of ExS_5_ vs. S_8_ condition on delayed retrieval *d′*. The ExS_5_ vs. S_8_ condition significantly predicted stage N2 duration (*B* = −0.928, *SE* = 0.076, *p* < .001), which, in turn, significantly predicted delayed retrieval *d′* (*B* = −0.616, *SE* = 0.330, *p* = .007). The total effect of experimental condition on delayed retrieval *d′* was not significant (*B* = −0.224, *SE* = 0.17, *p* = .183), but the direct effect was statistically significant (*B* = −0.795, *SE* = 0.80, *p* = .030). The 95% bootstrap CI for the indirect effect did not include zero (0.571, *SE* = 0.287, 95% bootstrap CI (0.008, 1.136)) (Table S6), indicating that the exercise-associated changes in stage N2 sleep duration may also contribute to memory benefits. The coefficients for the other exploratory mediation models are reported in Table S6.

### Moderation of memory effects of acute exercise by sleep architecture

Exploratory moderation analyses also showed that both the SWS percentage (interaction effect: *B* = 0.105, *SE* = 0.043, *p* = .018) and duration of SWS (*B* = 0.041, *SE* = 0.017, *p* = .0204; Table S7) significantly moderated the relationship between the ExS_5_ vs. S_5_ condition and delayed retrieval *d′* (Table S7), indicating that the memory benefit of exercise in sleep-restricted participants was higher in those with higher percentage and duration of SWS.

## Discussion

This study demonstrates that a single evening session of moderate-intensity exercise may buffer against sleep-restriction–induced memory impairment. Memory performance in the exercise plus sleep restriction group was comparable to that of participants with a full night of sleep, despite a 4-hour reduction in sleep opportunity. This sharply contrasts with the sedentary plus sleep restriction condition, which showed marked memory decline.

In line with our hypothesis, an acute evening session of moderate-intensity cardiovascular exercise before a night of sleep restriction partially mitigated the memory deficits induced by bleep loss on long-term declarative memory performance (i.e. delayed retrieval discriminability index, *d′*) in the ExS_5_ condition, relative to both the average (S_8_) and restricted (S_5_) sleep conditions. Acute exercise promoted sleep-dependent memory consolidation, evidenced by a greater difference in *d′* score in the ExS_5_ relative to S_5_, yet comparable to S_8_. Furthermore, the memory performance of ExS_5_ was comparable to that of the ExS_8_ condition, suggesting that acute exercise before sleep restriction can enhance memory performance to a level similar to that observed under normal sleep opportunity. As also hypothesized, the effect of the ExS_5_ versus S_8_ condition on long-term memory performance was statistically mediated by the percentage of SWS.

These findings reinforce our earlier work [48]. Our previous HIIT study [48] lacked objective sleep assessments (e.g. PSG or actigraphy) and objective monitoring of exercise intensity. The present study addressed these limitations by using PSG-verified sleep architecture, individualized VO_2max_ testing, and continuous heart rate monitoring during the exercise session, providing stronger evidence for the role of exercise in memory protection in sleep restriction.

The current findings, together with our prior work [48], suggest that the memory buffering effect of acute evening exercise on memory deficits under sleep restriction may vary by exercise intensity. In our previous study, HIIT intervention was associated with partial attenuation of sleep-restriction-related memory deficits relative to an average sleep condition, yet comparable to the sleep restriction without exercise condition. In contrast, the present study found that moderate-intensity cycling was associated with memory performance in the ExS_5_ group that was comparable to the S_8_ group and significantly better than the S_5_ group. The interaction effect size in the current study (partial η^2^ = 0.06) was larger than the effect size observed in our prior HIIT study (partial η^2^ = 0.033) [48], though direct statistical comparison across studies is not possible.

These patterns are consistent with the inverted-U hypothesis, which posits that moderate-intensity exercise yields optimal cognitive benefits compared with low- or high-intensity exercise [75, 76]. According to this hypothesis, moderate-intensity exercise may optimally engage arousal, attention, and neurotrophic signaling (e.g. BDNF), whereas low-intensity exercise may be insufficient to trigger these effects and high-intensity exercise may induce fatigue or stress that counteracts cognitive benefits [75, 76]. In line with this hypothesis, our previous HIIT study [48] showed modest partial protection against sleep-restriction-related memory impairment, whereas the moderate-intensity protocol in the present study was associated with memory performance approaching levels observed under normal sleep conditions. However, direct experimental comparison of different exercise intensities within a single study is needed to confirm this pattern.

In the present study, we also examined whether SWS percentage and, exploratorily, other sleep stages statistically mediated or partially accounted for the relationship between the experimental conditions and memory performance. We found that SWS percentage statistically mediated long-term memory performance (delayed retrieval *d′*) in the ExS_5_ versus S_8_ condition. This finding aligns with prior research demonstrating the central role of SWS in declarative memory processes [1–4]. Slow-wave sleep is most robustly associated with declarative memory consolidation, particularly during early-night, SWS-rich sleep, which preferentially supports hippocampal-dependent memory processes compared to later-night, REM-predominant sleep [77]. Additionally, SWS percentage positively moderated the relationship between the ExS_5_ versus S_5_ condition and long-term memory performance, further supporting its role in memory retention. The finding that SWS emerged as both a mediator (ExS_5_ vs. S_8_) and a moderator (ExS_5_ vs. S_5_) suggests that SWS may influence memory through two complementary pathways. As a state-like factor, SWS operated as a dynamic mechanism: exercise acutely enhanced SWS quality, which in turn improved memory consolidation. As a trait-like factor, SWS operated as a moderator—where individuals with higher SWS showed better cognitive performance under sleep restriction. These roles are likely not mutually exclusive; rather, SWS may function simultaneously as an acute mediator of exercise effects and a stable individual characteristic that influences susceptibility to sleep-loss-related memory deficits [78].

Despite the significantly higher SWS observed in both the S_5_ and ExS_5_ conditions relative to the S_8_ group, memory performance remained poorer in the S_5_ condition. This underscores the critical role of adequate sleep duration for optimal memory function [1, 2, 17, 79]. Our findings suggest that an elevated amount of SWS alone, as a compensatory response to acute sleep restriction, may be insufficient to restore memory performance in the absence of interventions (such as physical exercise) that are thought to protect the brain and cognitive functions against the effects of sleep loss [20]. Importantly, these findings further highlight the synergistic interaction between exercise and sleep in supporting memory processes, even under conditions of sleep restriction, as demonstrated by the ExS_5_ condition. Compared with the S_8_, the acute exercise intervention enhanced SWS in the ExS_5_ condition, consistent with prior studies [34, 80]. Acute exercise induces physiological changes in body temperature, autonomic activity, and metabolic functions that modulate key aspects of sleep architecture, essential for sleep-dependent memory consolidation and retention [20, 21, 26].

Notably, slow oscillations during SWS [10, 32, 33] and sleep spindles predominantly generated during stage N2 sleep, and SWS are key oscillatory mechanism required for sleep-dependent memory consolidation [34–37]. During SWS, SOs originating from prefrontal and medial temporal cortices coordinate the reactivation of hippocampal memory traces [5]. These SOs also group sleep spindles and hippocampal sharp-wave ripples, a coupling mechanism that facilitates long-term potentiation and systems consolidation [81]. Interventions that increase SWS and neuronal oscillations (e.g. SOs and spindle activity) during NREM sleep result in improved sleep-dependent memory consolidation [7–10]. Exercise has been shown to increase SOs and spindle amplitude in both animals and humans [21, 34]. Although our study did not analyze these microstructural events, the statistical mediation by SWS percentage is consistent with the possibility that exercise may enhance SOs–spindle coupling. Future studies should investigate whether exercise-induced changes in these oscillatory events mediate memory protection during sleep restriction.

Perhaps more importantly, our findings raised the question of why delayed retrieval performance differed between the S_5_ and ExS_5_ groups despite comparable immediate recall performance, suggesting that encoding and initial memory formation were statistically similar across conditions. The superior delayed retrieval performance observed in the ExS_5_ group relative to the S_5_ group is unlikely to be explained by differences in learning or rule acquisition during encoding. Rather, the beneficial effect of exercise emerged during the sleep period, implicating sleep-dependent consolidation mechanisms.

The Synaptic Homeostasis Hypothesis (SHY) [79, 82] offers a potential framework for explaining this pattern. The SHY proposes that sleep downscales synaptic strength potentiated during wakefulness (when learning occurs), thereby renormalizing neural circuits and improving the signal-to-noise ratio for subsequent retrieval [79, 82]. The SHY also suggests that more efficient synaptic downscaling during sleep, particularly SWS leads to better sleep-dependent memory consolidation and retention [79, 82]. In the context of our study, both S_5_ and ExS_5_ groups experienced significant sleep restriction, limiting the total time available for synaptic downscaling. However, our findings suggest that the acute moderate-intensity exercise may have promoted more synaptic downscaling process, possibly through its augmentation of SWS (which statistically mediated the memory effect), thereby preserving memory performance despite reduced sleep duration. Future studies directly testing synaptic markers are needed to confirm this interpretation.

Alternatively, our findings may also be interpreted within the framework of the sequential sleep hypothesis. According to this view, optimal memory consolidation requires the cyclic succession of both SWS and REM sleep, with SWS serving to select and weaken non-adaptive memory traces and REM sleep subsequently integrating the remaining traces into preexisting networks [83]. In our study, REM sleep was substantially reduced in sleep-restricted groups (minutes nearly halved), raising the possibility that exercise exerted its protective effect by compensating for lost REM function. However, our mediation analyses do not support this interpretation. REM percentage did not significantly mediate the effect of exercise on delayed recall, whereas SWS percentage did. Moreover, SWS percentage positively moderated the relationship between exercise and memory in the ExS_5_ vs. S_5_ comparison. These findings are more consistent with SWS rather than REM sleep replacement as the primary statistical pathway. Nevertheless, we cannot rule out interactive effects between SWS and REM sleep, and future studies manipulating both sleep stages are needed to disentangle their respective contributions to exercise-induced cognitive resilience under sleep restriction.

Another possible explanation for the compensatory effect observed in this study may be the ability of moderate-intensity exercise to engage neurophysiological mechanisms that counteract cognitive impairments [42]. Acute exercise elevates levels of BDNF and other neuronal growth factors in the hippocampus and related brain regions, stimulating neurogenesis, long-term potentiation and synaptic plasticity that are crucial for learning and memory [40, 42–44]. Even a single session of acute exercise (<1 hour) rapidly engages hippocampal neuroplasticity mechanisms. In humans, 10 minutes of mild exercise enhances dentate gyrus functional connectivity, with the magnitude of this effect predicting individual memory improvement [84]. A single 30-minute bout of moderate-intensity cycling increases both BDNF and anandamide, with BDNF specifically correlating with enhanced hippocampal memory representations and long-term retention [40]. Rodent studies confirm that a single exercise bout increases hippocampal BDNF expression within hours, independent of noradrenergic signaling and distinct from the cumulative effects of chronic training [85].

The temporal dynamics of these responses align with our study design. Evidence shows that BDNF expression peaks approximately 4-8 hours after acute exercise [86]. Our protocol, which included exercise at 7:00 p.m., encoding at 10:00 p.m., and bedtime at either at 11:00 p.m. (S_8_, ExS_8_) or 3:00 a.m. (S_5_, ExS_5_), falls within this window, allowing exercise-induced neurotrophic priming to coincide with memory encoding and subsequent sleep-dependent consolidation. These findings are consistent with our mediation results, suggesting that acute moderate-intensity exercise may prime hippocampal circuits for enhanced plasticity during sleep, with SWS percentage statistically accounting for this effect.

However, it is worth-noting that the timescale of our protocol, particularly the memory task (40-minute learning session, overnight memory testing), does not align with the weeks-long timeline required for adult hippocampal neurogenesis [87, 88]. Our study was not designed to capture neurogenesis. Rather, the observed associations are more plausibly explained by acute, BDNF-driven synaptic plasticity and long-term potentiation, which unfold within minutes to hours and are compatible with our timeline [40]. Thus, while learning can influence the survival of new neurons over longer periods [89], the present findings are best interpreted within the framework of rapid synaptic plasticity.

Our mediation analyses further support the contribution of stage N2 sleep to the consolidation and performance of declarative memories. Specifically, the duration of stage N2 sleep was positively associated with delayed retrieval memory performance in the ExS_5_ versus S_8_ comparison. Previous studies reported that certain forms of declarative memory (e.g. word-pair learning) rely mainly on stage N2 sleep and may remain unaffected by SWS deprivation [90]. Other evidence indicates that targeted memory reactivation can occur at comparable levels during both SWS and stage N2 sleep [77, 91]. Neural oscillatory activity characteristic of stage N2 sleep (i.e. spindles and delta waves) play a vital role in memory consolidation and enhancing declarative memory retention [77, 92]. Collectively, our findings, together with prior research [90], indicate that sleep-dependent memory consolidation is not exclusively reliant on SWS or REM, but may also depend on stage N2 sleep and its associated brain oscillations.

The differential mediation patterns for stage N2 sleep (minutes) and SWS percentage likely reflect the distinct functional roles of these sleep stages and the metrics that best capture their memory-relevant features under sleep restriction. Specifically, N2 sleep duration, but not N2 percentage, mediated the relationship between experimental condition and memory performance. This finding is consistent with the possibility that the absolute amount of N2 sleep may be particularly important for supporting memory processes associated with N2-related oscillatory activity (e.g. sleep spindles). In contrast, SWS percentage, but not SWS duration, emerged as a mediator. SWS percentage reflects the efficiency of compensatory SWS generation, which was elevated in both sleep-restricted groups, yet only ExS5 showed better memory performance. This suggests that exercise may have enhanced SWS quality (e.g. slow-wave activity power) rather than merely increasing its duration, which was capped by the short sleep opportunity [25, 34, 39, 80]. This is consistent with evidence that SWS intensity (density of slow oscillations) is more closely linked to memory consolidation than absolute amount of SWS [34]. Given the number of exploratory mediation and moderation tests conducted, there is an increased risk of Type I error, and that these exploratory observations should be interpreted cautiously. These findings should be considered hypothesis-generating and require replication studies to determine if these differential mediation patterns reflect distinct physiological mechanisms or chance variation.

Overall, our findings support prior experimental [48] and cross-sectional evidence that physical exercise may mitigate the cognitive deficits associated with poor sleep quality or insufficient sleep [49, 51, 52], while extending knowledge on the interactions between exercise and sleep on memory and cognition [45, 48, 49, 51, 52, 93]. Our findings also have important implications for the general public, given the growing prevalence of sleep loss globally. While our findings are promising, they were obtained in healthy young adults under controlled laboratory conditions. Thus, generalizability to clinical populations (e.g. insomnia, anxiety disorders, and depression) or occupational settings (e.g. shift workers, tactical personnel) requires empirical testing. Nevertheless, our results suggest that moderate-intensity exercise is a low-risk, accessible intervention that may buffer against some cognitive effects of sleep loss. Future studies should replicate these findings in more diverse and clinically relevant samples to specifically inform translational recommendations. Engaging in at least 30 minutes of moderate exercise has been shown to enhance brain electrophysiology, reflected by robust increases in SWS [34] and elevate BDNF levels, thereby supporting the preservation and restoration of cognitive functioning [42].

Our study has several ***strengths.*** To the best of our knowledge, this is the first well-controlled laboratory study to assess the extent to which an acute bout of moderate exercise may compensate for cognitive impairments induced by sleep restriction on long-term declarative memory. Acute sleep restriction targeting the first half of the night is an ecologically valid model of partial sleep deprivation that provides a reliable assessment of the neurobehavioral consequences of sleep loss [94]. We also used extensive, well controlled objective measures to assess exercise capacity, sleep, and cognition. Exercise capacity or cardiorespiratory fitness was directly measured to determine the individualized exercise intensity for the ExS_5_ and ExS_8_ groups, and exercise intensity was objectively monitored using heart rate monitors. We conducted baseline PSG to screen participants to rule out potential sleep disorders and experimental night’s sleep was measured using sleep electroencephalography (EEG). We also used wrist actigraphy to ensure consistent sleep/wake cycles during the week prior to the experimental night. Our sample included comparable numbers of both males and females, improving the generalizability of study findings.

Nevertheless, our study also has some ***limitations*** with implications for future research. First, cardiorespiratory fitness was not assessed in the sedentary groups, limiting objective confirmation of their baseline fitness. Second, the MEQ score (assessing chronotype) differed between the groups, with the exercise conditions reporting a tendency toward evening chronotype; however, MEQ was included as a covariate in the analyses, and it did not significantly influence the results. Third, Our between-subjects design limits within-person causal inference. Although groups were similar at baseline (Table 1), we cannot rule out unmeasured confounding. A within-subjects or crossover design would have allowed participants to serve as their own controls, reducing between-subjects variability and strengthening causal claims. However, such a design would have required each participant to complete four intensive experimental sessions (average sleep, sleep restriction, exercise + average sleep, exercise + sleep restriction), each including overnight PSG, evening exercise at 55%–60% VO₂max, a 40-minute learning session, and a delayed recall task. Based on pilot work, we anticipated that this would lead to high attrition and compromised data quality. Future studies using within-subjects or crossover designs are needed to replicate and extend our findings.

Fourth, because exercise occurred before encoding, we cannot disentangle effects on encoding versus consolidation. Improved initial learning after exercise could itself enhance subsequent SWS and memory. Thus, we interpreted our findings as showing that exercise is associated with better delayed memory performance after sleep restriction, with SWS as a mediator, without claiming exclusive consolidation effects. Fifth, by design, sleep-restricted participants remained awake for approximately 5 hours after encoding, whereas average-sleep participants had only a 1-hour wake interval. This differential wake period could introduce differences in interference or forgetting. However, both S_5_ and ExS_5_ had identical wake intervals yet differed significantly in memory performance, indicating that exercise—not wake interval accounted for the effect. Future studies using yoked designs (e.g. matched wake intervals across conditions) could further isolate sleep-specific from wake-related effects. Finally, future studies should also investigate the contributions of sleep micro-architecture or brain oscillations during sleep to the effects of physical exercise on memory impairments associated with sleep restriction or sleep loss.

**In conclusion**, the findings of this study show that a single bout of moderate-intensity evening cardiovascular exercise was associated with protection against the detrimental effects of acute sleep restriction on long-term declarative memory. Acute moderate-intensity exercise may promote sleep-dependent memory consolidation and long-term declarative memory performance in sleep restriction. The exercise plus sleep restriction condition resulted in significantly better memory performance than sleep restriction alone and comparably to the average sleep condition. The proportion of SWS and the duration of stage N2 sleep statistically mediated these effects. These findings are consistent with the possibility that acute exercise could support hippocampal-dependent declarative memory under sleep restriction, with potential implications for populations at risk of sleep loss due to occupational demands or sleep disorders.

## Supplementary Material

Supplementary_File_Frimpong_et_al_zsag235

## Data Availability

The data underlying this article will be shared on reasonable request to the corresponding author(s).
