# Supplementary material for: Slow-wave sleep mediates the compensatory effects of acute moderate-intensity exercise on declarative memory deficits in sleep restriction
Source: Sleep. 2026 Aug 26;49(11):zsag235. doi: 10.1093/sleep/zsag235 (PMC13747342; doi:10.1093/sleep/zsag235)
Supplement: Supplementary file 1 [file supplementary_file_frimpong_et_al_zsag235.docx]

**Slow-Wave Sleep Mediates the Compensatory Effects of Acute Moderate-Intensity Exercise on Declarative Memory Deficits in Sleep Restriction**

Emmanuel Frimpong^1,2,3,4*^, Melodee Mograss^1,2,3,5^, Jocelyn Dautet-Plourde^1,2,3^, Nicole Milbrath^1,3,5^, Francois Pointeau^1,2,3,4^, Manlu Xu^1,3,5^, Ngoc Long Khang Nguyen^1,3,5^, Tehila Zvionow^1,2,3^, Mylene Aubertin-Leheudre^4,6^, Louis Bherer^4,7^, Veronique Pepin^2,3,8^, Edwin Robertson^9^, Arsenio Paez^1,2,3,4*^, Thien Thanh Dang-Vu^1,2,3,4,5*^

**Affiliations:**

**^1^**Sleep, Cognition & Neuroimaging Laboratory, Concordia University, Montreal, QC, Canada.

**^2^**Department of Health, Kinesiology & Applied Physiology, Concordia University, Montreal, QC, Canada.

**^3^**School of Health, Concordia University, Montreal, QC, Canada.

**^4^**Centre de Recherche de l’Institut Universitaire de Gériatrie de Montréal, QC, Canada.

**^5^**Department of Psychology, Concordia University, Montreal, QC, Canada.

**^6^**Département des Sciences de l’activité physique, GRAPA, Université du Québec à Montréal, QC, Canada.

**^7^**Department of Medicine and Centre de recherche de l’Institut de cardiologie de Montréal, Université de Montréal, QC, Canada.

**^8^**Centre de recherche, CIUSSS du Nord-de l’Île-de-Montréal, Montréal, QC, Canada.

**^9^**School of Psychology and Neuroscience, University of Glasgow, Glasgow, United Kingdom.

*** Corresponding authors:**

Emmanuel Frimpong; Arsenio Paez & Thien Thanh Dang-Vu

***Address for correspondence***:

Sleep, Cognition & Neuroimaging (SCN) Laboratory, Department of Health, Kinesiology and Applied Physiology/PERFORM Centre, Concordia University, Montreal, QC, H4B 1R6, Canada.

Tel.: +1 (514)848-2424, ext. 3364

Emails: emmanuel.frimpong@mail.concordia.ca (EF); arsenio.paez@concordia.ca (AP) & tt.dangvu@concordia.ca (TTDV)

**SUPPLEMENTARY RESULTS**

**Supplementary Figures**

**Figure S1: Comparisons of immediate retrieval discriminability index between groups**. S_8_: average sleep only (8.5 h/night, *n* = 23), S_5_: restricted sleep only (4.5 h/night, n = 22), ExS_5_: exercise before restricted sleep (n = 22), ExS_8_: exercise before average sleep opportunity (*n* = 21). *d'*: discriminability index. Means ± SD.

**ns**: not significant, *p* > 0.05.

♦diamonds indicate the data points.

**Alt-text:**

Plunger plots with individual data points showing mean immediate discriminability index (d′) for the four experimental groups: S_8_ (average sleep only, n = 23), S_5_ (restricted sleep only, n = 22), ExS_5_ (exercise before restricted sleep, n = 22), and ExS_8_ (exercise before average sleep opportunity, n = 21). All groups show similar means with overlapping error bars (standard deviation, SD), and no significant differences are indicated between groups. Diamonds indicate the individual data points.

**Figure S2: Comparisons of the difference in the discriminability index (delayed – immediate retrieval d') between the groups**. S_8_: average sleep only (8.5 h/night, *n* = 23), S_5_: restricted sleep only (4.5 h/night, *n* = 22), ExS_5_: exercise before restricted sleep (n = 22), ExS_8_: exercise before average sleep opportunity (*n* = 21).

**ns**: not significant, *p* > 0.05.

*******p <* 0.001.

♦diamonds indicate the data points.

**Alt-text:**

Plunger plots with individual data points showing the difference in discriminability index (delayed retrieval minus immediate retrieval d′) for the four experimental groups: S_8_ (average sleep only, n = 23), S_5_ (restricted sleep only, n = 22), ExS_5_ (exercise before restricted sleep, n = 22), and ExS_8_ (exercise before average sleep opportunity, n = 21). The S_8_ (golden brown color), ExS_5_ (blue color) and ExS_8_ (green color) groups show comparable positive difference, indicating similar sleep-dependent memory consolidation. The S_5_ group (red color) show a negative difference, indicating poorest sleep-dependent memory consolidation. Error bars represent standard deviations. Significant differences (Bonferroni-adjusted) are indicated by asterisks between the groups. Diamonds indicate the individual data points.

**Figure S3:** **Comparing immediate retrieval face-name retrieval accuracy between groups**. S_8_: average sleep only (8.5 h/night, *n* = 23), S_5_: restricted sleep only (4.5 h/night, *n* = 22), ExS_5_: exercise before restricted sleep (n = 22), ExS_8_: exercise before average sleep opportunity (*n* = 21).

**ns**: not significant, *p* > 0.05.

♦diamonds indicate the data points.

**Alt-text:**

Plunger plots with individual data points showing mean immediate face-name retrieval accuracy (proportion correct) for the four experimental groups: S_8_ (average sleep only, n = 23), S_5_ (restricted sleep only, n = 22), ExS_5_ (exercise before restricted sleep, n = 22), and ExS_8_ (exercise before average sleep opportunity, n = 21). All groups show similar means with overlapping error bars (standard deviation, SD), and no significant differences are indicated between groups. Diamonds indicate the individual data points.

**Figure S4: Comparisons of the immediate retrieval motivation, performance, performance difficulty ratings and reaction time between the groups**. ***A***: motivation, ***B***: perceived performance, ***C***: performance difficulty, ***D***: sleepiness assessed with KSS, ***D***: reaction time, KSS: Karolinska Sleepiness Scale. S_8_: average sleep only (8.5 h/night, n = 23), S_5_: restricted sleep only (4.5 h/night, n = 22), ExS_5_: exercise before restricted sleep (n = 22), ExS_8_: exercise before average sleep opportunity (*n* = 21). Means ± SEM. For motivation, perceived performance, and task difficulty: 0 = low, 10 = high. For KSS: 1 = extremely alert, 9 = very sleepy.

*Significant.

**Alt-text:**

Panel of five bar graphs (A–E) comparing immediate retrieval performance rating measures and reaction time across the four groups: S_8_ (average sleep only, n = 23), S_5_ (restricted sleep only, n = 22), ExS_5_ (exercise before restricted sleep, n = 22), and ExS_8_ (exercise before average sleep opportunity, n = 21). Panels A (motivation), B (perceived performance) and C (performance difficulty) show similar ratings across groups. Panel D (sleepiness, assessed with KSS) shows higher ratings in the ExS_5_ group compared to ExS_8_ (p = 0.019). Panel E (reaction time) shows similar values across groups. Error bars represent standard deviations. Diamonds indicate individual data points. For Panel A (motivation), Panel B (perceived performance), and Panel C (performance difficulty): 0 means low, and 10 means high.

**Figure S5: Comparisons of the delayed retrieval motivation, performance, performance difficulty ratings and reaction time between the groups**. ***A***: motivation, ***B***: perceived performance, ***C***: performance difficulty, ***D***: sleepiness assessed with KSS, ***E***: reaction time, KSS: Karolinska Sleepiness Scale. S_8_: average sleep only (8.5 h/night, n = 23), S_5_: restricted sleep only (4.5 h/night, n = 22), ExS_5_: exercise before restricted sleep (n = 22), ExS_8_: exercise before average sleep opportunity (*n* = 21). Means ± SEM. For motivation, perceived performance, and task difficulty: 0 = low, 10 = high. For KSS: 1 = extremely alert, 9 = very sleepy.

*Significant.

**Alt-text:**

Panel of five bar graphs (A–E) comparing delayed retrieval performance rating measures and reaction time across the four groups: S_8_ (average sleep only, n = 23), S_5_ (restricted sleep only, n = 22), ExS_5_ (exercise before restricted sleep, n = 22), and ExS_8_ (exercise before average sleep opportunity, n = 21). Panels A (motivation), B (perceived performance) and C (performance difficulty) show similar ratings across groups. Panel D (sleepiness, assessed with KSS) shows higher ratings in the S_5_ and ExS_5_ groups compared to S_8_ and ExS_8_. Sleepiness was higher in the S_5_ group than the S_8_ (p < 0.001) and ExS_8_ (p < 0.001), and also higher in the ExS_5_ than S_8_ (p = 0.039); but was comparable between S_5_ versus ExS_5_ and ExS_5_ versus ExS_8_ groups. Panel E (reaction time) shows similar values across groups. Error bars represent standard deviations. Diamonds indicate individual data points. For Panel A (motivation), Panel B (perceived performance), and Panel C (performance difficulty): 0 means low, and 10 means high.

**Supplementary Tables**

**Table S1: Study exclusion criteria and cut-off scores**

BAI: Beck Anxiety Inventory, BDI-II: Beck Depression Inventory, ESS: Epworth Sleepiness Scale, IPAQ: International Physical Activity Questionnaire, ISI: Insomnia Severity Index, OSA: Obstructive sleep apnea, PSQI: Pittsburgh Sleep Quality Index, SBQ: STOP-Bang Questionnaire, UNS: Ullanlinna Narcolepsy Scale.

*If the participant provided information pertinent to the exclusion criteria or exceeded the cut-off score threshold for the questionnaire items, they were ineligible to participate.

**Table S2: Results of 2×2 ANCOVA for delayed retrieval *d'* with immediate *d'* as an additional covariate**. *N = 88*. Exercise conditions (exercise vs. sedentary, *n* = 43), sleep conditions (average vs. restricted sleep, *n* = 45). S_8_: average sleep only (n = 23), S_5_: restricted sleep only (n = 22), ExS_5_: exercise before restricted sleep (n = 22), ExS_8_: exercise before average sleep opportunity (n = 21). *d'*: discriminability index, MEQ: Morningness-Eveningness Questionnaire, *η_p_^2^*: partial eta-squared, *SS*: Type III Sum of Squares, *df*: degree of freedom, *MS*: Mean Square.

**p* < 0.05.

**Table S3: 2×2 ANCOVA for delayed face-name retrieval accuracy with immediate retrieval *d'* as a covariate**. *N = 88*. Exercise conditions (exercise vs. sedentary, *n* = 43), sleep conditions (average vs. restricted sleep, *n* = 45). S_8_: average sleep only (n = 23), S_5_: restricted sleep only (n = 22), ExS_5_: exercise before restricted sleep (n = 22), ExS_8_: exercise before average sleep opportunity (n = 21). *d'*: discriminability index, MEQ: Morningness-Eveningness Questionnaire, *η_p_^2^*: partial eta-squared, *SS*: Type III Sum of Squares, *df*: degree of freedom, *MS*: Mean Square.

**p* < 0.05.

**Table S4: Post hoc Bonferroni comparisons following 2×2 ANCOVA for delayed fac-name retrieval accuracy**. *N = 88*. S_8_: average sleep only (n = 23), S_5_: restricted sleep only (n = 22), ExS_5_: exercise before restricted sleep (n = 22), ExS_8_: exercise before average sleep opportunity (n = 21). *d'*: discriminability index, MEQ: Morningness-Eveningness Questionnaire, *η_p_^2^*: partial eta-squared, Mean Diff: mean difference, SE: standard error, df: degree of freedom.

**p* < 0.05.

Table S5: Baseline sleep architecture.

*N*=88. S_8_: average sleep only, S_5_: restricted sleep only, ExS_5_: exercise before restricted sleep, ExS_8_: exercise before average sleep opportunity. SD: standard deviation, TIB: time in bed, TST: total sleep time, AWK: number of awakenings after sleep onset, SOL: sleep onset latency, SE: sleep efficiency, WASO: wake after sleep onset, NREM: percentage and duration of non-rapid eye movement sleep stages N1 and N2, SWS slow-wave sleep, REM: rapid eye movement sleep.

**Table S6:** **Exploratory mediation analyses of the effects of experimental conditions on delayed retrieval *d'* via sleep architecture.**

*N*=88. Mediation coefficients adjusted for MEQ score (chronotype score). Groups - S_8_: average sleep only (n = 23), S_5_: restricted sleep only (n = 22), ExS_5_: exercise before restricted sleep (n = 22), ExS_8_: exercise before average sleep opportunity (n = 21). Percentage and duration of non-rapid eye movement sleep (NREM) stages N1, N2, and N3; REM: rapid eye movement sleep. Percentage and duration of non-rapid eye movement sleep stages N1, N2, and N3, SWS slow-wave sleep), REM: percentage and duration of rapid eye movement sleep stage. *d'*: discriminability index at delayed retrieval, Coeff ***(a)***: mediation model coefficient for path *a* (conditions predicting mediators), Coeff ***(b)***: mediation model coefficient for path ***b*** (conditions and mediators predicting *d'*), *SE*: standard error, ***ab***: indirect effects—effects of conditions on d' through the mediators, CI: confidence interval, BootSE bootstrap standard error, BootLLCI lower limit of bootstrap confidence interval, BootULCI, upper limit of bootstrap confidence interval.

**Boldened CI**: significant mediation effects.

**Table S7: Exploratory moderation analyses of the effects of experimental conditions on delayed retrieval *d'***.

*N*=88. Moderation coefficients adjusted for MEQ score (chronotype score). S_8_: average sleep only (n = 23), S_5_: restricted sleep only (n = 22), ExS_5_: exercise before restricted sleep (n = 22), ExS_8_: exercise before average sleep opportunity (n = 21). Percentage and duration of non-rapid eye movement sleep (NREM) stages N1, N2, and N3; REM: rapid eye movement sleep. Percentage and duration of non-rapid eye movement sleep stages N1, N2, and N3, SWS (slow-wave sleep), REM: percentage and duration of rapid eye movement sleep stage. *d'*: discriminability index, SE: standard error, CI: confidence interval, LLCI: lower limit of confidence interval, ULCI: upper limit of confidence interval.

**p* < 0.05.

Supplementary figures

**Figure S1: Comparisons of immediate retrieval discriminability index between groups**. S_8_: average sleep only (8.5 h/night, *n* = 23), S_5_: restricted sleep only (4.5 h/night, n = 22), ExS_5_: exercise before restricted sleep (n = 22), ExS_8_: exercise before average sleep opportunity (*n* = 21). *d'*: discriminability index. Means ± SD.

**ns**: not significant, *p* > 0.05.

♦diamonds indicate the data points

**Alt-text:**

Plunger plots with individual data points showing mean immediate discriminability index (d′) for the four experimental groups: S_8_ (average sleep only, n = 23), S_5_ (restricted sleep only, n = 22), ExS_5_ (exercise before restricted sleep, n = 22), and ExS_8_ (exercise before average sleep opportunity, n = 21). All groups show similar means with overlapping error bars (standard deviation, SD), and no significant differences are indicated between groups. Diamonds indicate the individual data points.

**Figure S2: Comparisons of the difference in the discriminability index (delayed – immediate retrieval d') between the groups**. S_8_: average sleep only (8.5 h/night, *n* = 23), S_5_: restricted sleep only (4.5 h/night, *n* = 22), ExS_5_: exercise before restricted sleep (n = 22), ExS_8_: exercise before average sleep opportunity (*n* = 21).

**ns**: not significant, *p* > 0.05.

*******p <* 0.001.

♦diamonds indicate the data points.

**Alt-text:**

Plunger plots with individual data points showing the difference in discriminability index (delayed retrieval minus immediate retrieval d′) for the four experimental groups: S_8_ (average sleep only, n = 23), S_5_ (restricted sleep only, n = 22), ExS_5_ (exercise before restricted sleep, n = 22), and ExS_8_ (exercise before average sleep opportunity, n = 21). The S_8_ (golden brown color), ExS_5_ (blue color) and ExS_8_ (green color) groups show comparable positive difference, indicating similar sleep-dependent memory consolidation. The S_5_ group (red color) show a negative difference, indicating poorest sleep-dependent memory consolidation. Error bars represent standard deviations. Significant differences (Bonferroni-adjusted) are indicated by asterisks between the groups. Diamonds indicate the individual data points.

**Figure S3:** Comparing immediate face-name retrieval accuracy between groups. S_8_: average sleep only (8.5 h/night, *n* = 23), S_5_: restricted sleep only (4.5 h/night, *n* = 22), ExS_5_: exercise before restricted sleep (n = 22), ExS_8_: exercise before average sleep opportunity (*n* = 21).

**ns**: not significant, *p* > 0.05.

♦diamonds indicate the data points.

**Alt-text:**

Plunger plots with individual data points showing mean immediate face-name retrieval accuracy (proportion correct) for the four experimental groups: S_8_ (average sleep only, n = 23), S_5_ (restricted sleep only, n = 22), ExS_5_ (exercise before restricted sleep, n = 22), and ExS_8_ (exercise before average sleep opportunity, n = 21). All groups show similar means with overlapping error bars (standard deviation, SD), and no significant differences are indicated between groups. Diamonds indicate the individual data points.

**Figure S4: Comparisons of the immediate retrieval motivation, performance, performance difficulty ratings and reaction time between the groups**. ***A***: motivation, ***B***: perceived performance, ***C***: performance difficulty, ***D***: sleepiness assessed with KSS, ***D***: reaction time, KSS: Karolinska Sleepiness Scale. S_8_: average sleep only (8.5 h/night, n = 23), S_5_: restricted sleep only (4.5 h/night, n = 22), ExS_5_: exercise before restricted sleep (n = 22), ExS_8_: exercise before average sleep opportunity (*n* = 21). Means ± SD. For motivation, perceived performance, and task difficulty: 0 = low, 10 = high. For KSS: 1 = extremely alert, 9 = very sleepy.

*Significant.

**Alt-text:**

Panel of five bar graphs (A–E) comparing immediate retrieval performance rating measures and reaction time across the four groups: S_8_ (average sleep only, n = 23), S_5_ (restricted sleep only, n = 22), ExS_5_ (exercise before restricted sleep, n = 22), and ExS_8_ (exercise before average sleep opportunity, n = 21). Panels A (motivation), B (perceived performance) and C (performance difficulty) show similar ratings across groups. Panel D (sleepiness, assessed with KSS) shows higher ratings in the ExS_5_ group compared to ExS_8_ (p = 0.019). Panel E (reaction time) shows similar values across groups. Error bars represent standard deviations. Diamonds indicate individual data points. For Panel A (motivation), Panel B (perceived performance), and Panel C (performance difficulty): 0 means low, and 10 means high.

**Figure S5: Comparisons of the delayed retrieval motivation, performance, performance difficulty ratings and reaction time between the groups**. ***A***: motivation, ***B***: perceived performance, ***C***: performance difficulty, ***D***: sleepiness assessed with KSS, ***E***: reaction time, KSS: Karolinska Sleepiness Scale. S_8_: average sleep only (8.5 h/night, n = 23), S_5_: restricted sleep only (4.5 h/night, n = 22), ExS_5_: exercise before restricted sleep (n = 22), ExS_8_: exercise before average sleep opportunity (*n* = 21). Means ± SD. For motivation, perceived performance, and task difficulty: 0 = low, 10 = high. For KSS: 1 = extremely alert, 9 = very sleepy.

*Significant.

**Alt-text:**

Panel of five bar graphs (A–E) comparing delayed retrieval performance rating measures and reaction time across the four groups: S_8_ (average sleep only, n = 23), S_5_ (restricted sleep only, n = 22), ExS_5_ (exercise before restricted sleep, n = 22), and ExS_8_ (exercise before average sleep opportunity, n = 21). Panels A (motivation), B (perceived performance) and C (performance difficulty) show similar ratings across groups. Panel D (sleepiness, assessed with KSS) shows higher ratings in the S_5_ and ExS_5_ groups compared to S_8_ and ExS_8_. Sleepiness was higher in the S_5_ group than the S_8_ (p < 0.001) and ExS_8_ (p < 0.001), and also higher in the ExS_5_ than S_8_ (p = 0.039); but was comparable between S_5_ versus ExS_5_ and ExS_5_ versus ExS_8_ groups. Panel E (reaction time) shows similar values across groups. Error bars represent standard deviations. Diamonds indicate individual data points. For Panel A (motivation), Panel B (perceived performance), and Panel C (performance difficulty): 0 means low, and 10 means high.

Supplementary Tables

**Table S1.** Study exclusion criteria and cut-off scores

| Exclusion criteria | Cut-Off Scores for exclusion* |
| --- | --- |
| 1. Being outside the target age range (18-35 years old) |  |
| 2. Evidence of chronic medical or psychological disorders | BAI score >36  BDI-II score >20 |
| 3. Evidence of poor sleep quality or sleep disorders | ISI score > 14  SBQ score indicating high risk of OSA  UNS score > 14  Irregularities or abnormalities in sleep/wake cycles during actigraphy screening  PSQI >5 |
| 4. Excessive daytime sleepiness | ESS >10 |
| 5. Regular use of psychotropic or hypnotic medication | Self-report on sleep diary or during actigraphy and sleep diary confirmation |
| 6. Shift work or having travelled through more than one time zone in the past month |  |

BAI: Beck Anxiety Inventory, BDI-II: Beck Depression Inventory, ESS: Epworth Sleepiness Scale, IPAQ: International Physical Activity Questionnaire, ISI: Insomnia Severity Index, OSA: Obstructive sleep apnea, PSQI: Pittsburgh Sleep Quality Index, SBQ: STOP-Bang Questionnaire, UNS: Ullanlinna Narcolepsy Scale.

*If the participant provided information pertinent to the exclusion criteria or exceeded the cut-off score threshold for the questionnaire items, they were ineligible to participate.

**Table S2: Results of 2×2 ANCOVA for delayed retrieval *d'* with immediate *d'* as an additional covariate**

| **Source** | ***SS*** | ***df*** | ***MS*** | ***F*** | ***P*** | ***η_p_^2^*** |
| --- | --- | --- | --- | --- | --- | --- |
| Sex | 0.002 | 1 | 0.002 | 0.002 | 0.961 | 0.000 |
| MEQ score | 0.011 | 1 | 0.011 | 0.018 | 0.895 | 0.000 |
| Immediate *d'* | 65.778 | 1 | 65.778 | 101.129 | <0.001* | 0.565 |
| Exercise conditions | 2.843 | 1 | 2.843 | 4.372 | 0.040* | 0.053 |
| Sleep conditions | 14.940 | 1 | 14.940 | 22.969 | <0.001* | 0.227 |
| Exercise x sleep interaction | 4.645 | 1 | 4.645 | 7.142 | 0.009* | 0.084 |
| Error | 50.734 | 78 | 0.650 |  |  |  |
| Total | 136.538 | 85 |  |  |  |  |

*N = 88*. Exercise conditions (exercise vs. sedentary, *n* = 43), sleep conditions (average vs. restricted sleep, *n* = 45). S_8_: average sleep only (n = 23), S_5_: restricted sleep only (n = 22), ExS_5_: exercise before restricted sleep (n = 22), ExS_8_: exercise before average sleep opportunity (n = 21). *d'*: discriminability index, MEQ: Morningness-Eveningness Questionnaire, *η_p_^2^*: partial eta-squared, *SS*: Type III Sum of Squares, *df*: degree of freedom, *MS*: Mean Square.

**p* < 0.05.

**Table S3: 2×2 ANCOVA for delayed face-name retrieval accuracy with immediate retrieval *d'* as a covariate**

| **Source** | ***SS*** | ***df*** | ***MS*** | ***F*** | ***P*** | ***η_p_^2^*** |
| --- | --- | --- | --- | --- | --- | --- |
| Sex | 0.002 | 1 | 0.002 | 0.239 | 0.626 | 0.003 |
| MEQ score | 0.001 | 1 | 0.001 | 0.162 | 0.688 | 0.002 |
| Immediate retrieval face-name accuracy | 1.752 | 1 | 1.752 | 217.456 | <0.001* | 0.736 |
| Exercise conditions | 0.137 | 1 | 0.137 | 16.948 | <0.001* | 0.179 |
| Sleep conditions | 0.191 | 1 | 0.191 | 23.752 | <0.001* | 0.233 |
| Exercise x sleep interaction | 0.189 | 1 | 0.189 | 23.512 | <0.001* | 0.232 |
| Error | 0.628 | 78 | 0.008 |  |  |  |
| Total | 37.549 | 85 |  |  |  |  |

*N = 88*. Exercise conditions (exercise vs. sedentary, *n* = 43), sleep conditions (average vs. restricted sleep, *n* = 45). S_8_: average sleep only (n = 23), S_5_: restricted sleep only (n = 22), ExS_5_: exercise before restricted sleep (n = 22), ExS_8_: exercise before average sleep opportunity (n = 21). *d'*: discriminability index, MEQ: Morningness-Eveningness Questionnaire, *η_p_^2^*: partial eta-squared, *SS*: Type III Sum of Squares, *df*: degree of freedom, *MS*: Mean Square.

**p* < 0.05.

**Table S4: Post hoc Bonferroni comparisons following 2×2 ANOVA for delayed fac-name retrieval accuracy**

| **Comparison** | **Mean Diff** | **t-value** | **df** | ***p*** | **Cohen's d** |
| --- | --- | --- | --- | --- | --- |
| S_5_ vs. ExS_5_ | -0.151 | 2.75 | 79 | 0.008* | 0.62 |
| S_8_ vs. ExS_8_ | 0.028 | 0.50 | 79 | 0.617 | 0.11 |
| S_5_ vs. S_8_ | -0.165 | 3.17 | 79 | 0.002* | 0.71 |
| ExS_5_ vs. ExS_8_ | 0.013 | 0.24 | 79 | 0.812 | 0.05 |
| S_5_ vs. ExS_8_ | -0.138 | 2.46 | 79 | 0.009* | 0.55 |
| S_8_ vs. ExS_5_ | 0.015 | 0.28 | 79 | 1.000 | 0.06 |

*N = 88*. S_8_: average sleep only (n = 23), S_5_: restricted sleep only (n = 22), ExS_5_: exercise before restricted sleep (n = 22), ExS_8_: exercise before average sleep opportunity (n = 21). *d'*: discriminability index, MEQ: Morningness-Eveningness Questionnaire, *η_p_^2^*: partial eta-squared, Mean Diff: mean difference, SE: standard error, df: degree of freedom.

**p* < 0.05.

Table S5. Baseline sleep architecture.

| **Variable** | **S_8_ (*n* = 23)**  **Mean ± SD** | **S_5_ (*n* = 22)**  **Mean ± SD** | **ExS_5_ (*n* = 22)**  **Mean ± SD** | **ExS_8_ (*n* = 21)**  **Mean ± SD** | ***P*** |
| --- | --- | --- | --- | --- | --- |
| TIB (h) | 7.6 ± 0.8 | 7.7 ± 0.7 | 7.5 ± 0.8 | 7.8 ± 0.5 | 0.681 |
| TST (h) | 6.8 ± 1.0 | 6.6 ± 0.8 | 6.6 ± 0.7 | 7.1 ± 0.8 | 0.243 |
| SOL (min) | 17.1 ± 11.9 | 19.6 ± 14.8 | 20.1 ± 13.6 | 16.4 ± 12.9 | 0.746 |
| AWK (number) | 15.8 ± 9.9 | 16.8 ± 9.6 | 14.6 ± 5.9 | 13.5 ± 8.9 | 0.631 |
| WASO (min) | 47.3 ± 26.7 | 61.6 ± 38.0 | 48.9 ± 29.1 | 41.9 ± 27.2 | 0.179 |
| SE (%)^γ^ | 89.2 ± 6.9 | 86.1 ± 8.1 | 88.3 ± 5.9 | 90.5 ± 6.0 | 0.193 |
| N1 (% of TST) | 5.9 ± 3.6 | 6.4 ± 3.9 | 5.5 ± 3.7 | 5.7 ± 3.8 | 0.876 |
| N1 (min) | 22.7 ± 14.4 | 24.7 ± 14.8 | 20.4 ± 12.1 | 23.6 ± 15.8 | 0.780 |
| N2 (% of TST) | 51.4 ± 6.3 | 52.3 ± 8.6 | 48.8 ± 7.5 | 49.0 ± 5.6 | 0.271 |
| N2 (min) | 210.8 ± 44.5 | 205.9 ± 31.9 | 193.2 ± 3.4 | 200.2 ± 46.8 | 0.511 |
| SWS (% of TST) | 24.7 ± 6.2 | 24.1 ± 9.7 | 27.6 ± 8.9 | 25.2 ± 6.0 | 0.474 |
| SWS (min) | 99.8 ± 24.8 | 98.1 ± 45.5 | 109.0 ± 35.2 | 106.6 ± 28.6 | 0.678 |
| REM (% of TST) | 17.8 ± 4.9 | 17.2 ± 5.9 | 18.5 ± 5.4 | 20.1 ± 4.5 | 0.299 |
| REM (min) | 73.5 ± 24.5 | 69.2 ± 27.8 | 73.7 ± 24.1 | 85.0 ± 22.8 | 0.201 |
| REM Lat (min) | 89.3 ± 32.1 | 98.6 ± 33.3 | 104.1 ± 39.2 | 88.6 ± 21.5 | 0.319 |

*N*=88. S_8_: average sleep only, S_5_: restricted sleep only, ExS_5_: exercise before restricted sleep, ExS_8_: exercise before average sleep opportunity. SD: standard deviation, TIB: time in bed, TST: total sleep time, AWK: number of awakenings after sleep onset, SOL: sleep onset latency, SE: sleep efficiency, WASO: wake after sleep onset, NREM: percentage and duration of non-rapid eye movement sleep stages N1 and N2, SWS slow-wave sleep, REM: rapid eye movement sleep.

Table S6. Exploratory mediation analyses of the effects of experimental conditions on delayed retrieval d' via sleep architecture.

|  | **Mediators Regressed on Conditions** | | | | **Delayed Retrieval d' Regressed on Mediators & Conditions** | | | **Indirect Effects/Bootstrapping (95% Bootstrap CI)** | | | |
| --- | --- | --- | --- | --- | --- | --- | --- | --- | --- | --- | --- |
| **Conditions/Groups** |  | **Coeff (*a*)** | ***SE*** | ***P*** | **Coeff (*b*)** | ***SE*** | ***P*** | **Effect (*ab*)** | ***BootSE*** | **BootLLCI** | **BootULCI** |
| **ExS_5_ vs. S_8_** | N1 (%) | - 0.2963 | 0.1423 | 0.0324 | -0.0128 | 0.1842 | 0.9450 | 0.0038 | 0.0601 | -0.1307 | 0.1296 |
|  | N2 (%) | -0.2782 | 0.1580 | 0.0856 | -0.1973 | 0.1629 | 0.2329 | 0.0549 | 0.0582 | -0.0454 | 0.1882 |
|  | REM (%) | -0.1381 | 0.1280 | 0.2870 | 0.0053 | 0.2047 | 0.9794 | -0.0007 | 0.0369 | -0.0974 | 0.0592 |
|  | N1 (min) | -0.5442 | 0.1330 | <0.001 | -0.2154 | 0.2362 | 0.3678 | 0.1172 | 0.1567 | -0.1775 | 0.4384 |
|  | N2 (min) | -0.928 | 0.076 | <0.001 | -0.616 | 0.330 | 0.007 | 0.571 | 0.287 | **0.008** | **1.136** |
|  | SWS (min) | -0.5244 | 0.1215 | <0.001 | -0.2323 | 0.2910 | 0.4297 | 0.1218 | 0.1714 | -0.2282 | 0.4656 |
|  | REM (min) | -0.6364 | 0.0865 | <0.001 | -0.4641 | 0.3381 | 0.1782 | 0.2953 | 0.276 | -0.1943 | 0.7130 |
| **S_5_ vs. S_8_** | N1 (%) | -0.5795 | 0.3187 | 0.0079 | -1.1356 | 0.4061 | 0.0079 | -0.1264 | 0.1632 | -0.5569 | 0.1443 |
|  | N2 (%) | -0.6340 | 0.2840 | 0.0311 | 4.2734 | 2.0130 | 0.0405 | -2.7093 | 1.8730 | **-7.4386** | **-0.1969** |
|  | SWS (%) | 0.9255 | 0.2327 | <0.001 | 4.5750 | 2.1597 | 0.0409 | 4.2342 | 2.593 | **0.678** | **10.805** |
|  | REM (%) | -0.1427 | 0.2513 | 0.573 | 32.710 | 2.57 | 0.039 | -4.6689 | 9.7787 | -20.4245 | 11.2238 |
|  | N1 (min) | -1.0886 | 0.2730 | <0.001 | -0.1377 | 0.2980 | 0.6466 | 0.1499 | 0.3392 | -0.5342 | 0.8494 |
|  | N2 (min) | -1.9029 | 0.1415 | <0.001 | -1.2674 | 0.7006 | 0.0786 | 2.4117 | 1.1391 | **0.0589** | **4.5433** |
|  | SWS (min) | -1.0213 | 0.2285 | <0.001 | -0.5892 | 0.3795 | 0.1290 | 0.6018 | 0.3488 | -0.0842 | 1.2790 |
|  | REM (min) | -1.1927 | 0.1671 | <0.001 | -0.3059 | 0.4675 | 0.5169 | 0.3649 | 0.5668 | -0.9470 | 1.3045 |
| **ExS_8_ vs. S_8_** | N1 (%) | -0.1147 | 0.1178 | 0.3363 | 0.2816 | 0.1775 | 0.1207 | -0.0323 | 0.036 | -0.0951 | 0.0540 |
|  | N2 (%) | -0.1167 | 0.1136 | 0.3104 | -0.0765 | 0.1897 | 0.6891 | 0.0089 | 0.0275 | -0.0610 | 0.0538 |
|  | SWS (%) | 0.0213 | 0.0963 | 0.8259 | 0.1539 | 0.2227 | 0.4936 | 0.0033 | 0.0232 | -0.0442 | 0.0579 |
|  | REM (%) | 0.1889 | 0.1064 | 0.0835 | -0.2596 | 0.1985 | 0.0.1985 | -0.0490 | 0.0472 | -0.1552 | 0.0307 |
|  | N1 (min) | -0.1199 | 0.1280 | 0.3548 | 0.0652 | 0.1804 | 0.7198 | -0.0078 | 0.0406 | -0.0520 | 0.1180 |
|  | N2 (min) | -0.0736 | 0.0682 | 0.2871 | -0.9990 | 0.4147 | 0.0212 | 0.0735 | 0.0709 | -0.0370 | 0.2413 |
|  | SWS (min) | 0.0086 | 0.1114 | 0.9388 | -0.2757 | 0.2522 | 0.2816 | -0.0024 | 0.0428 | -0.0959 | 0 .0895 |
|  | REM (min) | 0.1495 | 0.0915 | 0.1103 | -0.6194 | 0.2441 | 0.0156 | -0.0926 | 0.0676 | -0.2477 | 0.0166 |
| **ExS_5_ vs. S_5_** | N1 (%) | -0.0003 | 0.2965 | 0.9992 | 0.1821 | 0.2009 | 0.3703 | -0.0001 | 0.0827 | -0.1994 | 0.1547 |
|  | N2 (%) | 0.0204 | 2.2917 | 0.9447 | -0.1077 | 0.2057 | 0.6036 | -0.0022 | 0.0630 | -0.1498 | 0.1245 |
|  | SWS (%) | 0.0997 | 0.3078 | 0.7477 | -0.0301 | 0.1956 | 0.8785 | -0.0030 | 0.0755 | -0.1741 | 0.1531 |
|  | REM (%) | -0.1711 | 0.2740 | 0.5360 | 0.0946 | 0.2192 | 0.6686 | -0.0162 | 0.0790 | -0.2316 | 0.0872 |
|  | N1 (min) | 0.0126 | 0.1725 | 0.9420 | 0.4467 | 0.4488 | 0.3264 | 0.0056 | 0.1175 | -0.2919 | 0.2194 |
|  | N2 (min) | 0.0111 | 0.1034 | 0.9148 | 0.1882 | 0.9361 | 0.8418 | 0.0021 | 0.1220 | -0.2016 | 0.3129 |
|  | SWS (min) | 0.0441 | 0.1781 | 0.8056 | 0.2528 | 0.6029 | 0.6775 | 0.0112 | 0.1287 | -0.3339 | 0.2120 |
|  | REM (min) | -0.0753 | 0.1247 | 0.5495 | 0.4237 | 0.5882 | 0.4760 | -0.0319 | 0.1066 | -0.3077 | 0.1310 |
| **ExS_5_ vs. ExS_8_** | N1 (%) | 0.4264 | 0.2960 | 0.1579 | 0.1753 | 0.1928 | 0.3692 | 0.0747 | 0.1168 | -0,0515 | 0.3950 |
|  | N2 (%) | 0.0569 | 0.3208 | 0.8602 | -0.0234 | 0.1799 | 0.8972 | -0.0013 | 0.0595 | -0.1569 | 0.1073 |
|  | SWS (%) | -0.8036 | 0.3406 | 0.0236 | 0.1483 | 0.1677 | 0.3821 | -0.1192 | 0.1352 | -0.3883 | 0.1690 |
|  | REM (%) | 0.8201 | 0.3116 | 0.0122 | -0.3097 | 0.1781 | 0.0904 | -0.2539 | 0.1566 | -0.5853 | 0.0259 |
|  | N1 (min) | 0.8536 | 0.2880 | 0.0052 | 1.1882 | 1.2154 | 0.3884 | 0.1606 | 0.1980 | -0.2801 | 0.5234 |
|  | N2 (min) | 1.5490 | 0.1670 | <0.001 | -0.3432 | 0.4829 | 0.4821 | -0.5317 | 0.8323 | -2.2243 | 1.1565 |
|  | SWS (min) | 1.1834 | 0.2846 | <0.001 | -0.0858 | 0.3053 | 0.7803 | -0.1016 | 0.4389 | -1.0338 | 0.8269 |
|  | REM (min) | 1.7421 | 0.2296 | <0.001 | -0.5596 | 0.2717 | 0.1829 | -0.9749 | 0.5788 | -2.1725 | 0.1928 |

*N*=88. Mediation coefficients adjusted for MEQ score (chronotype score). Groups - S_8_: average sleep only (n = 23), S_5_: restricted sleep only (n = 22), ExS_5_: exercise before restricted sleep (n = 22), ExS_8_: exercise before average sleep opportunity (n = 21). Percentage and duration of non-rapid eye movement sleep (NREM) stages N1, N2, and N3; REM: rapid eye movement sleep. Percentage and duration of non-rapid eye movement sleep stages N1, N2, and N3, SWS slow-wave sleep), REM: percentage and duration of rapid eye movement sleep stage. *d'*: discriminability index at delayed retrieval, Coeff ***(a)***: mediation model coefficient for path *a* (conditions predicting mediators), Coeff ***(b)***: mediation model coefficient for path ***b*** (conditions and mediators predicting *d'*), *SE*: standard error, ***ab***: indirect effects—effects of conditions on d' through the mediators, CI: confidence interval, BootSE bootstrap standard error, BootLLCI: lower limit of bootstrap confidence interval, BootULCI: upper limit of bootstrap confidence interval.

**Boldened CI**: significant indirect (mediation) effects.

**Table S7.** Exploratory moderation analyses of the effects of experimental conditions on delayed retrieval *d'*

|  | **Moderation** | | | | | | **Interaction** | | |
| --- | --- | --- | --- | --- | --- | --- | --- | --- | --- |
| **Conditions/Groups** | **Moderators** | **Coeff** | ***SE*** | **LLCI** | **ULCI** | ***P*** | ***Coeff*** | ***SE*** | ***P*** |
| **S_5_ vs. S_8_** | N1 (%) | -0.03 | 0.18 | -0.39 | 0.32 | 0.851 | 0.06 | 0.11 | 0.593 |
|  | N2 (%) | -0.04 | 0.08 | -0.20 | 0.12 | 0.612 | 0.03 | 0.05 | 0.845 |
|  | SWS (%) | 0.11 | 0.09 | -0.08 | 0.30 | 0.238 | -0.09 | 0.06 | 0.108 |
|  | REM (%) | -0.06 | 0.12 | -0.30 | 0.19 | 0.643 | 0.06 | 0.07 | 0.414 |
|  | N1 (min) | -0.03 | 0.05 | -0.13 | 0.07 | 0.596 | 0.03 | 0.04 | 0.411 |
|  | N2 (min) | -0.03 | 0.02 | -0.07 | 0.02 | 0.273 | 0.02 | 0.02 | 0.415 |
|  | SWS (min) | 0.03 | 0.02 | -0.01 | 0.07 | 0.211 | -0.03 | 0.02 | 0.105 |
|  | REM (min) | -0.03 | 0.03 | -0.08 | 0.03 | 0.282 | 0.03 | 0.02 | 0.189 |
| **ExS_5_ vs. S_8_** | N1 (%) | 0.06 | 0.11 | -0.15 | 0.28 | 0.568 | -0.03 | 0.05 | 0.534 |
|  | N2 (%) | -0.01 | 0.04 | -0.09 | 0.08 | 0.861 | -0.01 | 0.02 | 0.723 |
|  | SWS (%) | 0.01 | 0.06 | -0.10 | 0.13 | 0.814 | 0.01 | 0.02 | 0.820 |
|  | REM (%) | 0.01 | 0.07 | -0.14 | 0.16 | 0.872 | -0.01 | 0.03 | 0.782 |
|  | N1 (min) | 0.02 | 0.03 | -0.04 | 0.07 | 0.579 | -0.01 | 0.02 | 0.595 |
|  | N2 (min) | -0.01 | 0.11 | -0.04 | 0.01 | 0.281 | 0.001 | 0.01 | 0.779 |
|  | SWS (min) | -0.01 | 0.11 | -0.03 | 0.02 | 0.547 | 0.01 | 0.01 | 0.299 |
|  | REM (min) | -0.01 | 0.02 | -0.04 | 0.03 | 0.781 | 0.001 | 0.01 | 0.935 |
| **ExS_8_ vs. S_8_** | N1 (%) | -0.01 | 0.10 | -0.21 | 0.20 | 0.951 | 0.03 | 0.04 | 0.356 |
|  | N2 (%) | -0.03 | 0.04 | -0.12 | 0.06 | 0.485 | 0.02 | 0.02 | 0.325 |
|  | SWS (%) | 0.03 | 0.06 | -0.09 | 0.15 | 0.661 | -0.01 | 0.019 | 0.673 |
|  | REM (%) | 0.03 | 0.07 | -0.11 | 0.18 | 0.663 | -0.03 | 0.02 | 0.216 |
|  | N1 (min) | -0.001 | 0.02 | -0.05 | 0.05 | 0.973 | 0.01 | 0.01 | 0.424 |
|  | N2 (min) | -0.02 | 0.01 | -0.04 | 0.01 | 0.171 | 0.004 | 0.004 | 0.244 |
|  | SWS (min) | 0.001 | 0.01 | -0.02 | 0.02 | 0.937 | -0.001 | 0.004 | 0.748 |
|  | REM (min) | 0.001 | 0.01 | -0.03 | 0.03 | 0.973 | -0.004 | 0.005 | 0.344 |
| **ExS_5_ vs. S_5_** | N1 (%) | 0.34 | 0.29 | -0.23 | 0.924 | 0.236 | -0.13 | 0.12 | 0.285 |
|  | N2 (%) | 0.09 | 0.14 | -0.19 | 0.37 | 0.511 | -0.04 | 0.05 | 0.447 |
|  | SWS (%) | -0.29 | 0.11 | -0.51 | -0.06 | 0.015* | 0.11 | 0.04 | 0.018* |
|  | REM (%) | 0.22 | 0.15 | -0.10 | 0.53 | 0.169 | -0.08 | 0.06 | 0.227 |
|  | N1 (min) | 0.14 | 0.12 | -0.10 | 0.38 | 0.237 | -0.05 | 0.05 | 0.286 |
|  | N2 (min) | 0.03 | 0.05 | -0.08 | 0.14 | 0.597 | -0.01 | 0.02 | 0.567 |
|  | SWS (min) | -0.11 | 0.04 | -0.20 | -0.02 | 0.018* | 0.04 | 0.02 | 0.020* |
|  | REM (min) | 0.07 | 0.06 | -0.04 | 0.19 | 0.211 | -0.02 | 0.02 | 0.292 |
| **ExS_8_ vs. S_5_** | N1 (%) | 0.05 | 0.18 | -0.32 | 0.42 | 0.797 | 0.02 | 0.06 | 0.731 |
|  | N2 (%) | -0.01 | 0.10 | -0.21 | 0.19 | 0.912 | 0.01 | 0.03 | 0.718 |
|  | SWS (%) | -0.15 | 0.09 | -0.32 | 0.03 | 0.097 | 0.04 | 0.03 | 0.1.86 |
|  | REM (%) | 0.02 | 0.10 | 0.004 | 0.41 | 0.046* | -0.07 | 0.03 | 0.030* |
|  | N1 (min) | 0.05 | 0.07 | -0.-9 | 0.19 | 0.47 | -0.01 | 0.02 | 0.749 |
|  | N2 (min) | 0.01 | 0.04 | -0.07 | 0.08 | 0.83 | -0.001 | 0.01 | 0.891 |
|  | SWS (min) | -0.05 | 0.03 | -0.12 | 0.01 | 0.105 | 0.012 | 0.01 | 0.162 |
|  | REM (min) | 0.06 | 0.03 | -0.01 | 0.13 | 0.031* | -0.02 | 0.01 | 0.040* |
| **ExS_5_ vs. ExS_8_** | N1 (%) | -0.55 | 0.41 | -1.37 | 0.28 | 0.186 | 0.17 | 0.11 | 0.138 |
|  | N2 (%) | -0.22 | 0.16 | -0.54 | 0.11 | 0.184 | 0.06 | 0.05 | 0.175 |
|  | SWS (%) | 0.13 | 0.14 | -0.15 | 0.41 | 0.357 | -0.03 | 0.04 | 0.404 |
|  | REM (%) | 0.19 | 0.88 | -0.24 | 0.62 | 0.382 | -0.07 | 0.06 | 0.257 |
|  | N1 (min) | -0.13 | 0.15 | -0.43 | 0.16 | 0.371 | 0.04 | 0.04 | 0.305 |
|  | N2 (min) | -0.03 | 0.05 | -0.14 | 0.08 | 0.537 | 0.01 | 0.01 | 0.531 |
|  | SWS (min) | 0.06 | 0.05 | -0.03 | 0.16 | 0.199 | -0.02 | 0.01 | 0.198 |
|  | REM (min) | 0.04 | 0.07 | -0.10 | 0.18 | 0.560 | -0.01 | 0.02 | 0.431 |

*N*=88. Moderation coefficients adjusted for MEQ score (chronotype score). S_8_: average sleep only (n = 23), S_5_: restricted sleep only (n = 22), ExS_5_: exercise before restricted sleep (n = 22), ExS_8_: exercise before average sleep opportunity (n = 21). Percentage and duration of non-rapid eye movement sleep (NREM) stages N1, N2, and N3; REM: rapid eye movement sleep. Percentage and duration of non-rapid eye movement sleep stages N1, N2, and N3, SWS (slow-wave sleep), REM: percentage and duration of rapid eye movement sleep stage. *d'*: discriminability index, SE: standard error, CI: confidence interval, LLCI: lower limit of confidence interval, ULCI: upper limit of confidence interval.

**p* < 0.05.
